# Single-Cell Analysis Reveals Distinct Gene Expression and Heterogeneity in Male and Female Plasmodium falciparum Gametocytes

**DOI:** 10.1128/mSphere.00130-18

**Published:** 2018-04-11

**Authors:** Katelyn A. Walzer, Danielle M. Kubicki, Xiaohu Tang, Jen-Tsan Ashley Chi

**Affiliations:** a Department of Molecular Genetics and Microbiology, Duke University, Durham, North Carolina, USA; b Center for Genomic and Computational Biology, Duke University, Durham, North Carolina, USA; c Department of Biological Sciences, Michigan Technological University, Houghton, Michigan, USA; The Hebrew University

**Keywords:** single cell, RNA-FISH, Plasmodium falciparum, sexual development, gene expression

## Abstract

Sexual reproduction is an obligate step in the Plasmodium falciparum life cycle, with mature gametocytes being the only form of the parasite capable of human-to-mosquito transmission. Development of male and female gametocytes takes 9 to 12 days, and although more than 300 genes are thought to be specific to gametocytes, only a few have been postulated to be male or female specific. Because these genes are often expressed during late gametocyte stages and for some, male- or female-specific transcript expression is debated, the separation of male and female populations is technically challenging. To overcome these challenges, we have developed an unbiased single-cell approach to determine which transcripts are expressed in male versus female gametocytes. Using microfluidic technology, we isolated single mid- to late-stage gametocytes to compare the expression of 91 genes, including 87 gametocyte-specific genes, in 90 cells. Such analysis identified distinct gene clusters whose expression was associated with male, female, or all gametocytes. In addition, a small number of male gametocytes clustered separately from female gametocytes based on sex-specific expression independent of stage. Many female-enriched genes also exhibited stage-specific expression. RNA fluorescent *in situ* hybridization of male and female markers validated the mutually exclusive expression pattern of male and female transcripts in gametocytes. These analyses uncovered novel male and female markers that are expressed as early as stage III gametocytogenesis, providing further insight into *Plasmodium* sex-specific differentiation previously masked in population analyses. Our single-cell approach reveals the most robust markers for sex-specific differentiation in *Plasmodium* gametocytes. Such single-cell expression assays can be generalized to all eukaryotic pathogens.

**IMPORTANCE** Most human deaths that result from malaria are caused by the eukaryotic parasite Plasmodium falciparum. The only form of this parasite that is transmitted to the mosquito is the sexual form, called the gametocyte. The production of mature gametocytes can take up to 2 weeks and results in phenotypically distinct males and females, although what causes this gender-specific differentiation remains largely unknown. Here, we demonstrate the first use of microfluidic technology to capture single gametocytes and determine their temporal sex-specific gene expression in an unbiased manner. We were able to determine male or female identity of single cells based on the upregulation of gender-specific genes as early as mid-stage gametocytes. This analysis has revealed strong markers for male and female gametocyte differentiation that were previously concealed in population analyses. Similar single-cell analyses in eukaryotic pathogens using this method may uncover rare cell types and heterogeneity previously masked in population studies.

## Abstract (precis)

Most human deaths that result from malaria are caused by the eukaryotic parasite Plasmodium falciparum. The only form of this parasite that is transmitted to the mosquito is the sexual form, called the gametocyte. The production of mature gametocytes can take up to 2 weeks and results in phenotypically distinct males and females, although what causes this gender-specific differentiation remains largely unknown. Here, we demonstrate the first use of microfluidic technology to capture single gametocytes and determine their temporal sex-specific gene expression in an unbiased manner. We were able to determine male or female identity of single cells based on the upregulation of gender-specific genes as early as mid-stage gametocytes. This analysis has revealed strong markers for male and female gametocyte differentiation that were previously concealed in population analyses. Similar single-cell analyses in eukaryotic pathogens using this method may uncover rare cell types and heterogeneity previously masked in population studies.

## INTRODUCTION

Malaria remains a global health threat, with nearly 438,000 deaths and 214 million new cases in 2015 alone (1). The majority of these deaths are caused by Plasmodium falciparum, the most virulent of the *Plasmodium* species. The life cycle of P. falciparum is complex and includes multiple developmental stages in the human host and *Anopheles* mosquito vector. In humans, the parasite undergoes a 48-h intraerythrocytic developmental cycle (IDC) characterized by a highly synchronized and continuous cascade of gene expression, from merozoite erythrocyte invasion through cellular division (schizogony). This results in up to 32 new merozoites (2). Most of these parasites propagate asexually and maintain infection of host erythrocytes. A small proportion, around 5% but up to 30%  depending on strain and growth conditions, commits to a sexual fate and develops into gametocytes, which is critical for the transmission of malaria (3–5). The effective targeting of these sexually differentiated gametocytes may present therapeutic opportunities for human malaria, but much remains unknown about the molecular determinants of commitment and differentiation to a male or female sexual form.

All merozoites from a single schizont are committed to an asexual or sexual fate, with sexual merozoites developing into mature male and female gametocytes over a 9- to 12-day period (6). These gametocytes mature through five morphologically distinct stages (7). Only stage V gametocytes circulate in the peripheral blood and are available for transmission to the mosquito, where sexual reproduction takes place. P. falciparum gametocyte populations are female biased, with approximately one male for every five females depending on the clone (8, 9), although the molecular basis of male and female fate is unknown. While a single schizont will produce either all male gametocytes or all female gametocytes (10, 11), these parasites are morphologically indistinguishable until stage IV gametocytogenesis, when females can be differentiated from males by their smaller nucleus and blue Giemsa staining pattern (in contrast to the pink stain of male gametocytes) (12). Upon ingestion by a mosquito, stage V males undergo exflagellation, during which a single male divides to form eight flagellated microgametes. A single female gametocyte gives rise to a single immotile female macrogamete that is fertilized by a flagellated male gamete, forming a diploid zygote that develops into an ookinete.

Although at least 300 genes are considered to be gametocyte specific in P. falciparum, their roles in male and female development have not yet been fully defined (13–18). Plasmodium berghei and P. falciparum gender-specific flow sorting studies have revealed late-stage markers for male and female gametocytes, but these studies are based on specific reporter genes and are therefore biased for late stages (19–21). In particular, the recent flow sorting study using P. falciparum represents the first transcriptome analysis of male and female gametocytes (20). However, the gender-specific expression of some genes is still debated (22). We hypothesize that some of the controversies about gender-specific expression may result from reliance on population analyses of mixed gametocytes that include multiple differentiation stages and temporal changes in gene expression. These issues can best be resolved using single-cell isolation and expression analyses at distinct time points to unequivocally decipher sex-specific transcripts that may ultimately determine male or female fate. Recently, single-cell RNA sequencing revealed that sexually committed schizonts have a distinct program of gene expression (23). Here, we describe our efforts using a single-cell approach to define male and female gametocyte gene expression in an unbiased manner. Our study incorporates the first use of the Fluidigm C1 system for microfluidic capture of single gametocytes, followed by real-time PCR (RT-PCR) quantitation of their sex-specific expression of gametocyte genes on the Biomark HD system. The analysis of stage III through stage V gametocytes separates parasites by gender rather than stage and reveals a number of new candidate genes for male and female development. Additionally, a large female population reveals unexpected cellular heterogeneity among single cells, previously undetected on a population level. Therefore, our study highlights the power of single-cell transcriptome analysis in dissecting the sex-specific gene expression of P. falciparum.

## RESULTS AND DISCUSSION

### Male and female gene expression is anticorrelative at the single-cell level.

While a number of genes have been previously described as gametocyte specific or enriched, very few studies have elucidated what makes a parasite male or female, particularly at the transcript level. In addition, previous analyses have focused on the bulk population, which may include multiple stages and temporal changes in gene expression not fully considered during experimental design or data interpretation. Therefore, we applied single-cell isolation and expression analyses of enriched gametocytes to define male and female gametocyte gene expression in an unbiased manner. We optimized the Fluidigm C1 protocol for eukaryotic pathogens by adding a DNase I treatment to the cell lysis step to avoid genomic DNA as a confounding factor, a necessary modification for genes containing small introns. We selected a panel of gametocyte-specific transcripts (see Table S1 in the supplemental material) for single-cell quantitative PCR (qPCR) based on several criteria. First, we included the genes found by microarray analysis (13, 14) and mass spectrometry (15) to be highly upregulated during gametocytogenesis. Second, we prioritized for genes conserved among *Plasmodium* species, including P. berghei. Third, we prioritized for genes exported during gametocytogenesis (16). Finally, we selected genes previously shown to be involved in early gametocytogenesis (17). From these data, we selected 87 gametocyte-specific genes, 2 housekeeping genes, and 2 asexual markers to design primers for qPCR.

10.1128/mSphere.00130-18.5 TABLE S1  Primers used in Biomark single-cell qPCR. Download TABLE S1, PDF file, 0.1 MB. Copyright © 2018 Walzer et al. 2018 Walzer et al. This content is distributed under the terms of the Creative Commons Attribution 4.0 International license.

**FIG 1:**
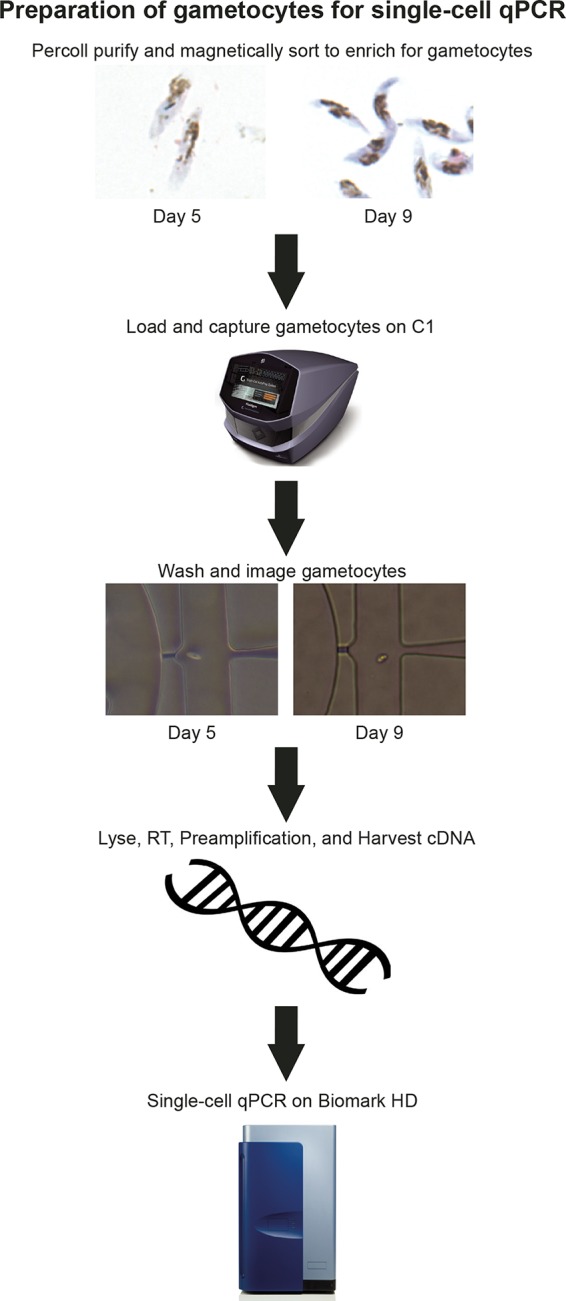
Preparation of gametocytes for single-cell qPCR. Stage III gametocytes were collected on day 5, and stage IV-V gametocytes were collected on day 9. These mid- to late-stage gametocytes were separated from rings, early gametocytes, and uninfected red blood cells (RBCs) by a 40/70% Percoll density gradient and were further purified from uninfected RBCs by MACS. These gametocytes were loaded onto a C1 IFC for single-cell capture. After each well was checked for the presence of a single cell, the single gametocytes were lysed, and cDNA was synthesized and preamplified with primers for qPCR. This harvested cDNA was then used for single-cell qPCR on the Biomark HD system, which can run up to 9,216 parallel reactions on a 96.96 Dynamic Array IFC.

Gametocytes were enriched in culture by treatment with 50 mM *N*-acetylglucosamine for 72 h and were collected for single-cell analyses on days 5 (stage III) and 9 (stage IV-V). Gametocyte-infected erythrocytes were purified from uninfected erythrocytes by a 40/70% Percoll density gradient and magnetic-activated cell sorting (MACS) before being loaded onto the Fluidigm C1 integrated fluidic circuit (IFC) for single-cell capture (Fig. 1). Before further processing, each microfluidic chamber was visually inspected under an inverted microscope to validate the presence of a single parasite. The rate of capture was approximately 76% for stage III gametocytes and 92% for stage IV-V gametocytes. We selected 90 gametocytes (45 from each day captured) to perform multiplex RT-PCR profiling of 87 gametocyte-specific genes, one cell of which we later discovered was asexual. Additionally, we included negative (no template and no reverse transcriptase) and positive (bulk population) controls as well as synthetic RNA spike-ins to provide normalization standards and technical controls.

**FIG 2:**
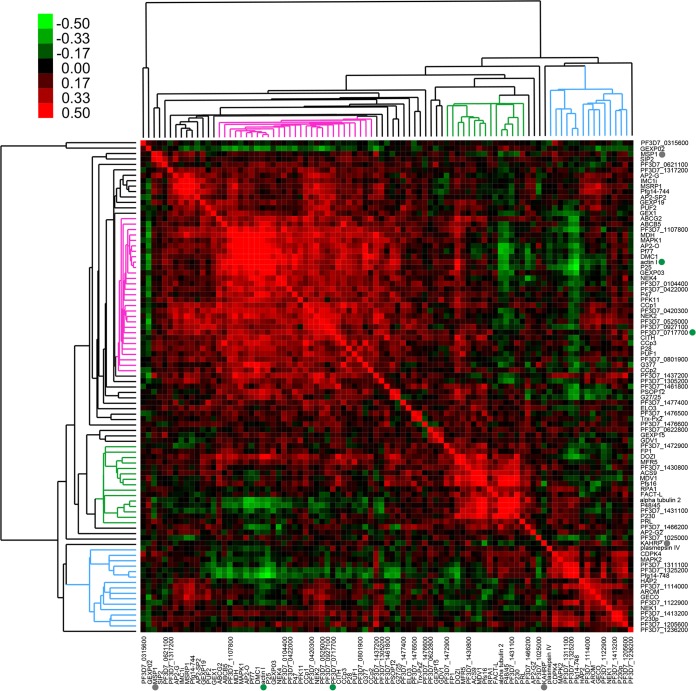
Pearson correlation of gene expression identifies distinct groups of male- and female-enriched genes. Eighty-seven gametocyte-specific genes, two housekeeping genes (solid green circles), and two asexual markers (solid gray circles) were used in single-cell qPCR and subsequent analyses. The raw *C*_*T*_ values for single-cell expression in 89 gametocytes, 1 asexual cell, and 2 population controls were normalized to the average of the RNA spike-ins before Pearson correlation analysis was performed. The pink cluster denotes female-associated genes, while the blue cluster denotes male-associated genes. The green cluster includes genes that are constitutively expressed in all samples.

To group the genes based on the similarity in expression patterns, we calculated the Pearson correlation of all genes used in this study based on their expression among 89 gametocytes, one asexual cell, and two population controls, with one control for each day of capture. Then we used hierarchical clustering to group the genes based on their correlations of expression patterns. Interestingly, we found that all the genes were grouped into three major clusters (Fig. 2). When the genes comprising each cluster were examined, we found that these clusters were arranged in a sex-specific manner according to previously described male and female markers (Fig. 2). One gene cluster (pink in Fig. 2) contained many genes previously recognized to encode markers for female gametocytogenesis, including G377 (24–26), Pf77 (27), P25 (28), P47 (29), and ABCG2 (30). In addition, NEK2 and NEK4 were also found in this cluster, suggesting their female-specific expression. Consistent with this concept, the loss of NEK2 or NEK4 in P. berghei abolished ookinete formation, a developmental defect linked to the female gametocyte (19, 31, 32). Similarly, when DMC1, another gene in the female cluster, was knocked out in P. berghei, oocyst formation was greatly reduced (33). The coclustering of DMC1 with other female genes suggests that this defect is female linked. Furthermore, these genes, along with CCp1 and CCp3, were found to be upregulated in gametocytes expressing P47-green fluorescent protein (GFP) in a recently published study (20), further supporting their association with female gametocytes.

AP2 transcription factors are important for all developmental stages of *Plasmodium*, and recently, AP2-G was found to be critical for sexual commitment in the small population of parasites that commit to gametocytogenesis (17, 34). Therefore, eight putative AP2 genes were included in this analysis, including five that are translationally repressed by the gametocyte-associated DOZI (development of zygote inhibited) complex: AP2-O, SIP2, AP2-SP2, PF3D7_1305200, and PF3D7_1107800 (35, 36). Of these genes, only AP2-O and PF3D7_1107800 strongly cocluster with female-specific markers, which suggests that they are transcribed in female gametocytes and play a later role in the zygote.

Additionally, several other genes in the putative female cluster have not been previously characterized for their female expression. These novel candidate female-enriched genes include ABCB5, malate dehydrogenase (MDH), GEXP03, and mitogen-activated protein kinase 1 (MAPK1). MAPK1 stands out as a potential regulator of female gametocytogenesis because it is only one of two mitogen-activated protein kinases identified in P. falciparum, the other being MAPK2 (37–39). In particular, MAPK2 is essential for male gametogenesis in P. berghei (19, 40, 41), and its male-specific expression pattern holds true in our data set as well.

Pearson correlation shows a cluster containing male-specific markers (blue in Fig. 2). Besides MAPK2, these markers include HAP2 (42, 43), NEK1 (44), CDPK4 (45), P230p (46), PF3D7_1413200 (47), PF3D7_1122900 (19, 47), and PF3D7_1114000 (19, 47), the last two of which code for dynein chains. PF3D7_1311100, PF3D7_1325200, and Pfg14-748, whose functions are unknown, are mostly negatively correlated with female-specific genes, such as ABCG2, AP2-O, Pf77, and P25. This indicates that these three genes may be the most strongly male-specific markers. Pfg14-748 was previously described to be an exported protein expressed in early gametocytes (16, 48), but its male-specific expression in our single-cell data suggests that it may play an unexpected role in male gametocytes. Plasmepsin IV also unexpectedly clusters as male specific. Expressed during trophozoite and schizont stages, plasmepsin IV is an aspartic protease that degrades host hemoglobin within the food vacuole (49). It has also been shown to be expressed in the Plasmodium gallinaceum ookinete and may play a role in invasion of the mosquito midgut or in ookinete to oocyst development (50). Our data suggest that in P. falciparum, plasmepsin IV may be more important in male gametocytes and has an undetermined male-specific role. In support of our single-cell analysis, the genes in this male cluster are also upregulated in gametocytes expressing PF3D7_1023100-GFP (dynein heavy chain), supporting their association with male gametocytes (20).

In addition to the male- and female-specific clusters, there is a distinct cluster (green in Fig. 2) that represents genes that are constitutively expressed across both male and female parasites. This includes known sex-specific marker Pfs16, expressed throughout gametocytogenesis (46). It also includes a number of genes that were previously described to be specific to males or enriched in males, including MDV1, FACT-L, alpha tubulin 2, and P48/45 (29, 51–54). While it is possible that these genes are enriched in certain stages of male differentiation, particularly alpha tubulin 2 and P48/45, they may not be the most robust markers for male and female specificity. Male-specific markers PF3D7_1311100, PF3D7_1325200, and Pfg14-748 may be better suited to identifying male gametocytes and the abundance of stage V male gametocytes in infected human populations.

### Single male parasites cluster separately from female parasites based on their distinct transcriptional profiles.

**FIG 3:**
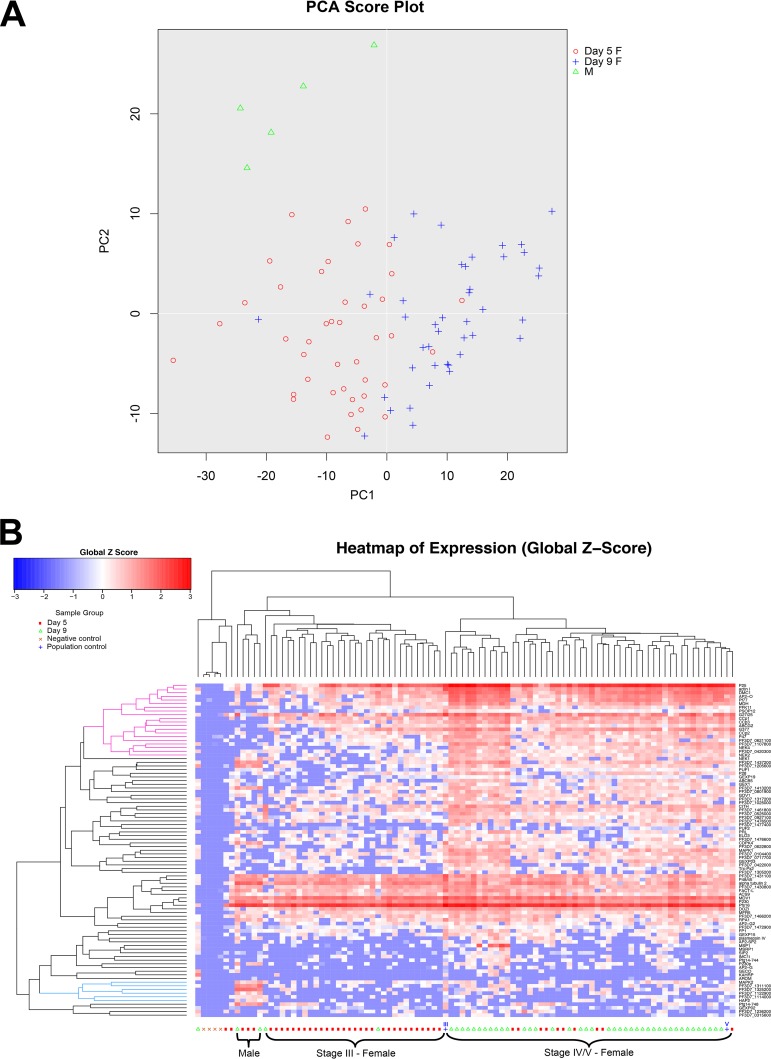
Gene expression separates male and female single gametocytes into distinct populations. (A) Principal component analysis (PCA) separates single gametocytes into male (M) and female (F) populations based on their gene expression. Furthermore, the female population is generally separated by stage, indicating differences in gene expression in mid- and late-stage female gametocytes. PC1, principal component 1. (B) The SINGuLAR Analysis Toolset 3.0 was used to perform unbiased hierarchical clustering on 89 mid- to late-stage gametocytes, 1 asexual cell, and 6 control samples, including positive and negative controls. Eighty-seven gametocyte-specific genes, two housekeeping genes, and two asexual markers were used in this analysis. Genes are clustered based on the Pearson method, while samples are clustered by the Euclidean method. Complete linkage was used to find similar clusters. The blue cluster denotes genes that are higher (enriched) in males, while the pink cluster denotes genes enriched in females.

Next, we used principal component analysis (PCA) (Fig. 3A) and hierarchical clustering (Fig. 3B and Fig. S1 and S2) to group individual parasites based on their gene expression pattern. Interestingly, the parasites were clearly arranged based on gender and specific differentiation stages. PCA analysis clearly separated the male gametocytes from all female gametocytes (Fig. 3A). The female gametocytes were largely separated into two groups, captured at middle (III) and late (IV-V) stages. These results were also recapitulated in hierarchical clustering with males captured on days 5 and 9 clustering together and females separating by their stage of maturation (Fig. 3B). Similar parasite groupings were also obtained by different selection, filtering, and clustering algorithms, indicating the robustness of the clustering based on gene expression (Fig. S1). Additionally, there were fewer males in the sample size than expected. The 3d7a strain was previously reported to produce 12.8% male gametocytes (22), but the single-cell qPCR data set includes only 5 males, 84 females, and, unexpectedly, 1 asexual cell (5.6% male [Fig. 3B]). Even with such a small number of male parasites, their distinct gene expression allows us to separate them from the outnumbering female parasites.

10.1128/mSphere.00130-18.1 FIG S1  Evaluation of the reproducibility of the parasite grouping based on hierarchical clustering. The gene expression of parasites was selected, filtered, and clustered using different filtering criteria and clustering algorithms as indicated. All male gametocytes were grouped in the same cluster. Female gametocytes were largely grouped by differentiation stage by all criteria and algorithms tested. Download FIG S1, PDF file, 0.3 MB. Copyright © 2018 Walzer et al. 2018 Walzer et al. This content is distributed under the terms of the Creative Commons Attribution 4.0 International license.

10.1128/mSphere.00130-18.2 FIG S2  Gene expression separates male and female gametocytes into distinct populations. The SINGuLAR Analysis Toolset 3.0 was used to perform unbiased hierarchical clustering on 45 gametocytes collected on day 5 (A) and 44  gametocytes collected on day 9 plus one asexual cell (B). Both analyses include three control samples. Eighty-seven gametocyte-specific genes, two housekeeping genes, and two asexual markers were used in these analyses. Download FIG S2, PDF file, 1.5 MB. Copyright © 2018 Walzer et al. 2018 Walzer et al. This content is distributed under the terms of the Creative Commons Attribution 4.0 International license.

**FIG 4:**
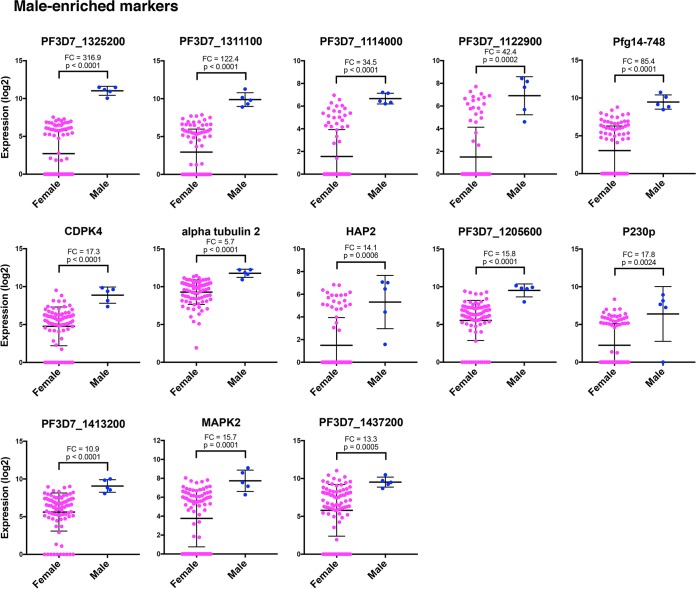
Individual genes exhibit distinct male-enriched expression patterns. Dot plots represent the most robust markers for male and female sexual differentiation ordered by PCA gene scores, with the topmost left gene being the most informative. The Mann-Whitney test was used to determine the significance of gene expression differences in males versus females. Values for the female and male gametocytes are shown as means ± standard deviations (error bars). The mean fold change (FC) in gene expression between males and females and the *P* value are shown for each gene.

These data indicate that a number of gender-specific markers can be used to differentiate the two sexes from stage III through stage V. In line with the Pearson correlation analysis (Fig. 2), male gametocytes show high expression of PF3D7_1311100, PF3D7_1325200, and PF3D7_1114000 (Fig. 3, 4, and S2). This includes high expression in all male cells, regardless of stage, indicating that males and females can be separated by their gene expression as early as stage III. In particular, PF3D7_1325200 is a putative lactate dehydrogenase, and its significantly high expression in male gametocytes suggests that it might be important for male development. PF3D7_1311100 is a putative meiosis-specific nuclear structural protein 1, which was found in mice to be essential for normal assembly of the sperm flagella (55). This suggests a role for this gene in male development and exflagellation.

Other genes that cluster with male single gametocytes include PF3D7_1122900, Pfg14-748, HAP2, and MAPK2. These genes are independently reported to be associated with male gametocytes in PF3D7_1023100-GFP (dynein heavy chain) expressing gametocytes (20). Both dynein chain male markers used in this study, PF3D7_1114000 and PF3D7_1122900, strongly associate as male (Fig. 3 and 4). However, it should be noted that these genes are expressed in a few putative female gametocytes as well (Fig. 4). Genes found to be male specific in both Pearson correlation and single-cell hierarchical clustering and expressed in male gametocytes at significantly higher levels than females according to the Mann-Whitney test are listed in Table 1. These genes are identified as the most robust male gametocyte markers.

**TABLE 1:** The most robust markers for male and female gene expression

Gene ID^a^	Gene name and description	Cluster
PF3D7_1031000	P25, 25-kDa ookinete surface antigen precursor	Female
PF3D7_1246200	Actin I	Female
PF3D7_0816800	DMC1, meiotic recombination protein, putative	Female
PF3D7_1143100	AP2-O, AP2 domain transcription factor, putative	Female
PF3D7_0621400	Pf77	Female
PF3D7_1128300	PFK11, ATP-dependent 6-phosphofructokinase	Female
PF3D7_1475500	CCp1, LCCL domain-containing protein	Female
PF3D7_1407000	CCp3, LCCL domain-containing protein	Female
PF3D7_1426500	ABCG2, ABC transporter G family member 2	Female
PF3D7_1250100	G377, osmiophilic body protein G377	Female
PF3D7_1346800	P47, 6-cysteine protein	Female
PF3D7_1107800	ApiAP2, AP2 domain transcription factor, putative	Female
PF3D7_0719200	NEK4, NIMA-related kinase 4	Female
PF3D7_1455800	CCp2, LCCL domain-containing protein	Female
PF3D7_1113900	MAPK2, mitogen-activated protein kinase 2	Male
PF3D7_1311100	Meiosis-specific nuclear structural protein 1, putative	Male
PF3D7_1325200	Lactate dehydrogenase, putative	Male
PF3D7_1122900	Dynein heavy chain, putative	Male
PF3D7_1114000	Dynein light chain Tctex-type, putative	Male
PF3D7_1014200	HAP2, male gamete fusion factor, putative	Male

a ID, identifier, or accession number, in PlasmoDB database.

These robust male markers show strong concordance with P. falciparum male-specific transcript expression in a separate study of sorted male and female gametocyte populations (20) (Fig. S3). Compared to the flow-sorted male transcriptome, all of the male markers identified in our study are more highly upregulated in male gametocytes than female gametocytes, many dramatically so and all more than 7.9-fold higher in males than in females (20) (Fig. S3). In particular, PF3D7_1311100 and PF3D7_1325200 stood out for their high male expression in both data sets, with mean fold changes of 122.4 and 316.9 in our data set alone (Fig. 4 and S3).

10.1128/mSphere.00130-18.3 FIG S3  Transcript levels of genes from male and female populations sorted in Lasonder et al. (20). The transcript levels, represented by normalized reads per kilobase per million (RPKM) values, were log transformed for the most robust male and female markers from our single-cell analyses (Table 1). Because the expression values were not mean centered, the transcript abundance is indicated by the intensity of the color scale. RPKM values below 1 were removed as indicated by the color gray in the heatmap. Download FIG S3, PDF file, 0.2 MB. Copyright © 2018 Walzer et al. 2018 Walzer et al. This content is distributed under the terms of the Creative Commons Attribution 4.0 International license.

**FIG 5:**
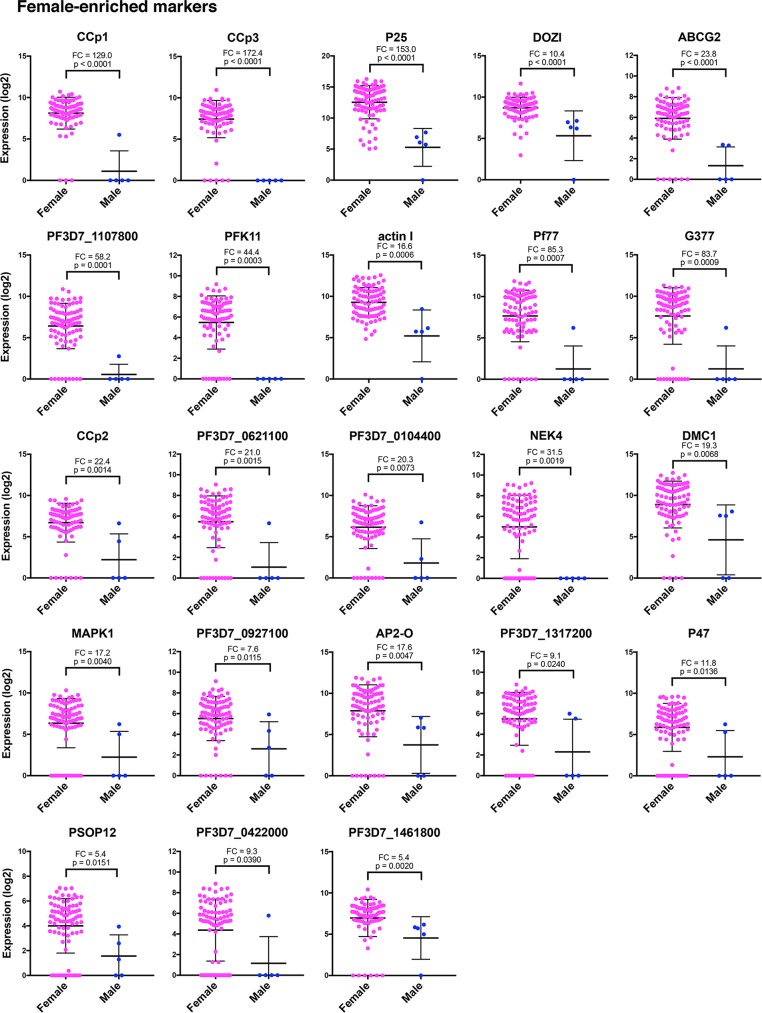
Individual genes exhibit distinct female-enriched expression patterns. Dot plots represent the most robust markers for male and female sexual differentiation ordered by PCA gene scores, with the topmost left gene being the most informative. The Mann-Whitney test was used to determine the significance of gene expression differences in males versus females. Values for the female and male gametocytes are shown as means ± standard deviations (error bars). The mean fold change (FC) in gene expression between males and females and the *P* value are shown for each gene.

To validate the expression of these genes in male gametocytes and to increase the male sample size, we performed RNA fluorescent *in situ* hybridization (RNA-FISH). Pfs16 was used to mark gametocyte-specific cells, which were then evaluated for their expression of male and female markers. One male marker was tested in combination with a female marker to determine male- and female-specific transcript expression. PF3D7_1311100 and PF3D7_1325200 were tested as new markers for male gametocytes, as their functions are unknown. We also included MAPK2 to assess male-specific expression, as this gene was previously shown to be upregulated in P. falciparum and P. berghei male populations (19–21). These markers were compared against female markers P25 and CCp1, which are both highly upregulated in the female-specific transcriptome and our single-cell qPCR data (20) (Fig. 3B, 5, and S3). Furthermore, although P25 has been used as a marker to identify gametocytes from field isolates (56), it was recently tested and shown to be female specific (28), consistent with our expression profiling.

**FIG 6:**
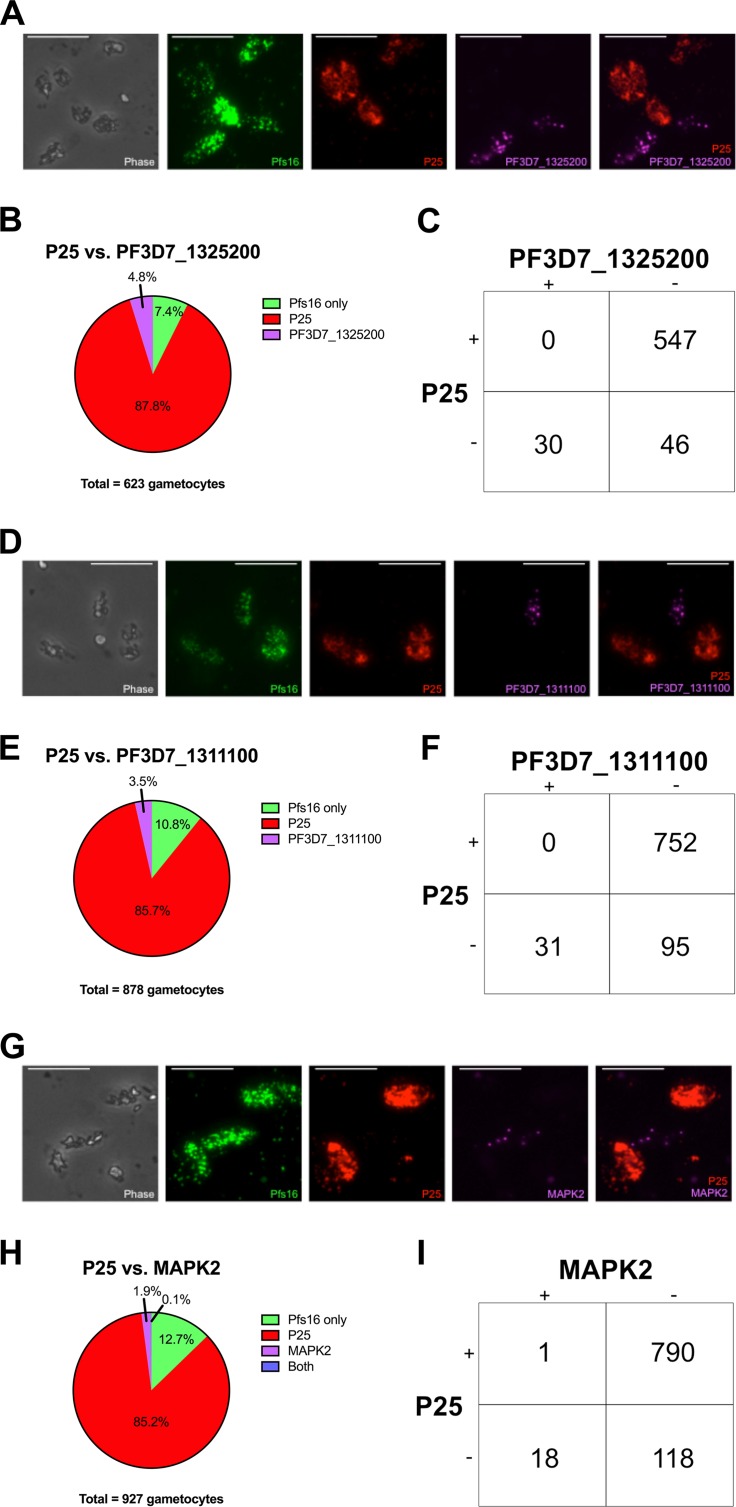
RNA-FISH validation of male and female markers. (A) Representative images of gametocytes expressing Pfs16 (gametocyte [green]), P25 (female [red]), and PF3D7_1325200 (male [purple]). Bars, 5 µm. (B) Pie chart showing the distribution of P25 and PF3D7_1325200 expression in Pfs16+ cells. (C) Diagram representing mutually exclusive expression of P25 and PF3D7_1325200. (D) Representative images of gametocytes expressing Pfs16 (gametocyte [green]), P25 (female [red]), and PF3D7_1311100 (male [purple]). (E) Pie chart showing the distribution of P25 and PF3D7_1311100 expression in Pfs16+ cells. (F) Diagram representing mutually exclusive expression of P25 and PF3D7_1311100. (G) Representative images of gametocytes expressing Pfs16 (gametocyte [green]), P25 (female [red]), and MAPK2 (male [purple]). (H) Pie chart showing the distribution of P25 and MAPK2 expression in Pfs16+ cells. (I) Diagram representing mutually exclusive expression of P25 and MAPK2.

Our RNA-FISH results clearly show that male and female gametocytes differentially express the tested genes (Fig. 6 and S4). In particular, PF3D7_1311100, PF3D7_1325200, and P25 were expressed at high levels and showed mutually exclusive expression in more than 600 gametocytes tested for each experiment (Fig. 6). The number of gametocytes expressing PF3D7_1325200 and PF3D7_1311100 was also low (4.8% and 3.5%, respectively), similar to the results obtained for single-cell qPCR. MAPK2 was expressed in even fewer cells (1.9%) with significant variations among male single cells, not seen in bulk cell analyses. Additionally, some gametocyte cells did not express either male or female marker. This proportion changed for each male marker tested (7.4%, 10.8%, and 12.7% [Fig. 6B, E, and H, respectively]), suggesting that some of these gametocytes are either earlier stage males/females or an undefined population.

10.1128/mSphere.00130-18.4 FIG S4  RNA-FISH validation of male and female markers. (A) Representative images of gametocytes expressing Pfs16, CCp1, and PF3D7_1325200. Bars, 5 µm. (B) Pfs16+ cells were counted for their expression of CCp1 and PF3D7_1325200. (C) Diagram representing mutually exclusive expression of CCp1 and PF3D7_1325200. (D) Representative images of gametocytes expressing Pfs16, CCp1, and PF3D7_1311100. (E) Pfs16+ cells were counted for their expression of CCp1 and PF3D7_1311100. (F) Diagram representing mutually exclusive expression of CCp1 and PF3D7_1311100. (G) Representative images of gametocytes expressing Pfs16, CCp1, and MAPK2. (H) Pfs16+ cells were counted for their expression of CCp1 and MAPK2. (I) Diagram representing mutually exclusive expression of CCp1 and MAPK2. Download FIG S4, PDF file, 0.6 MB. Copyright © 2018 Walzer et al. 2018 Walzer et al. This content is distributed under the terms of the Creative Commons Attribution 4.0 International license.

A number of other reported candidate male-specific genes also show enriched expression in male single cells, but they do not always cluster with male-specific genes, suggesting a more complicated expression pattern than gender identity (Fig. 3, 4, and S2). These include CDPK4, alpha tubulin 2, P230p, and PF3D7_1413200. In particular, CDPK4 is an essential regulator of cell cycle progression in the male gametocyte and is necessary for parasite transmission to the mosquito (45). Male single cells generally show higher expression of this gene than females, but many females still express it (Fig. 4). A similar pattern is seen in alpha tubulin 2, P230p, and PF3D7_1413200, which suggests that their roles in male differentiation are more complex and may result from posttranscriptional regulations.

**FIG 7:**
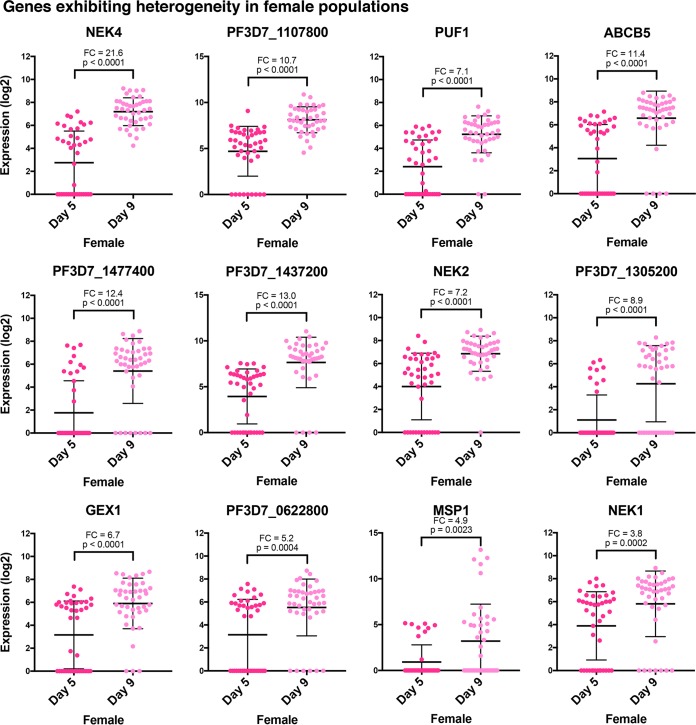
Individual genes exhibit distinct stage-specific expression among female gametocytes. Dot plots, which are ordered by PCA gene scores, show that a number of genes are more highly expressed in gametocytes collected on day 9 than in gametocytes collected on day 5. Many genes also have a bimodal distribution of gene expression on day 5. The Mann-Whitney test was used to determine the significance of gene expression differences in samples collected on day 5 versus day 9. The error bars represent the mean with the standard deviation. The mean fold change (FC) in gene expression in female gametocytes collected on day 9 and day 5 is shown for each gene.

We also wished to identify genes that are highly enriched in female gametocytes. We found that CCp1, CCp3, and P25 were the best markers to identify female gametocytes from male gametocytes. These three genes were highly expressed in stage III to stage V females (Fig. 3 and 5). CCp1 and P25 were additionally validated as female specific using RNA-FISH (Fig. 6 and S4). NEK4 is also strongly female specific, although it does not appear to be expressed until stages IV and V, so it is best used as a late-stage female marker (Fig. 3, 5, and 7). PF3D7_1107800, a putative AP2 transcription factor, strongly correlates as female specific and has increased expression in stage IV-V females relative to stage III females (Fig. 7). This suggests that this transcription factor may be used as a marker for late-stage female gametocytes. Further efforts to identify the targets of PF3D7_1107800 may reveal distinct biological processes in the zygote that result from a female-specific transcriptional program.

Other female markers that are significantly upregulated in female gametocytes include ABCG2, P77, G377, CCp2, AP2-O, and P47. PFK11, an ATP-dependent 6-phosphofructokinase, is also female specific, but its role in gametocytogenesis is unknown. PF3D7_0621100 also has an unknown function. PSOP12, another gene in our cluster that is enriched in females, encodes the putative secreted ookinete protein 12. PSOP12 is expressed during the sexual and ookinete stages of P. berghei and localizes to the surface of male and female gametocytes, male and female gametes, and ookinetes. Antibodies raised against PSOP12 demonstrate significant transmission-blocking capacity both *in vivo* and *in vitro* (57). Nevertheless, the role of PSOP12 in gametocytogenesis remains unknown, and our data suggest that it may be more important for female development in P. falciparum.

These genes are also independently validated as female-enriched by their upregulation in P47-GFP-expressing gametocytes (20). Of note, P47 associates with female gametocytes, but it is not expressed in all cells, including some stage III gametocytes (Fig. 3 and 5). This indicates that P47 likely starts being transcribed during stage III, so previous flow sorting based on P47 expression may not capture all of the stage III females. The most robust markers of female gametocytogenesis resulting from Pearson correlation, single-cell hierarchical clustering, and the Mann-Whitney test are listed in Table 1.

When looking more closely at the most robust female markers, it is clear that their high expression in female gametocytes is in strong agreement with P. falciparum female-specific transcript expression on a population level, with all markers being expressed at least 5.7-fold higher in females than in males (20) (Fig. S3). The most differentially expressed genes between females and males are ABCG2, Pf77, and P25, with 44.3-, 39.5-, and 35.6-fold changes, respectively. In our single-cell data set, these same genes showed 23.8-, 85.3-, and 153.0-fold changes, all significantly higher than males (Fig. 5).

Actin I is also a strong marker for female gametocytogenesis, and this was unexpected (Fig. 3 and 5). Actin I and PF3D7_0717700, a serine-tRNA ligase, were both chosen as constitutive controls and were expected to show relatively uniform expression across all cells. Instead, actin I was enriched 16.6-fold in our data set and 13.2-fold in flow-sorted P47-GFP females (20) (Fig. S3), while PF3D7_0717700 is not even expressed in all female single cells. This shows the variability of gene expression revealed on the single-cell level and exhibits the need to identify markers that can be used in all parasite life cycle stages.

Additionally, there are other genes, like DOZI and MAPK1, which do not cluster as female but show significantly higher expression in single female gametocytes (Fig. 5). This suggests that either transcript level or posttranscriptional regulation is important for the female-specific functions of these genes. In particular, DOZI is involved in the translational repression of transcripts during gametocytogenesis, with RNA immunoprecipitation studies in P. berghei identifying 731 transcripts that are associated with either DOZI or another RNA-binding protein called CITH (35, 58, 59). More recently, this translational repression was shown to be important in female P. falciparum gametocytes, with some repressed transcripts not being translated until the oocyst or sporozoite stage (20). This includes PF3D7_1107800 and AP2-O, two transcription factors found to be enriched in females in our study. It is possible that the higher transcript levels of DOZI in females are regulated in a sex-specific manner, a possibility that will need to be further investigated.

Our single-cell data also indicate that gene expression is stage specific, at least in female gametocytes. Eighty-four female gametocytes generally cluster by the day of differentiation, indicating the synchrony of the induced gametocyte population as well as the gradual change in gene expression during mid- to late-stage gametocyte development (Fig. 3B). Stage III gametocytes cluster together, with many female cells not expressing a number of the genes used in this single-cell analysis. Among those genes expressed in the female population, a number of genes show a bimodal distribution during stage III with an increase in expression during later stages (Fig. 7). This indicates that these genes are turning on during stage III with further enhanced expression during differentiation into stage V.

The rightmost cluster in Fig. 3B shows higher expression in some genes than the adjacent cluster, while the middle cluster of the heatmap shows the highest overall gene expression. This indicates a relative increase in gene expression of female-enriched genes over time, as the female gametocyte prepares for entry into the mosquito midgut and transcribes many genes that are translationally repressed until later stages in the mosquito. In particular, NEK2, NEK4, and GEX1 in our study show increased expression in later stages (Fig. 7). Their mutants from previous studies showed defects postfertilization, specifically in the ookinete or oocyst (31, 32, 60). Therefore, high expression of some genes in stage V female gametocytes may be most important for downstream functions in the mosquito, such as fertilization, meiosis, and sporogony. The increase in expression of AP2 factors, such as PF3D7_1107800 and PF3D7_1305200, may also serve the same purpose, as these transcription factors were shown to be translationally repressed by DOZI (35, 36). The potential functions of these AP2 factors in reproduction need to be further investigated.

Notably, transcript expression of merozoite surface protein 1 (MSP1) was seen in a few of the stage V female gametocytes. This is an unexpected finding, as MSP1 was used as an asexual marker to test for contaminating trophozoites (Fig. 3 and 7). MSP1 is the most abundant surface protein of merozoites and plays a role in parasite egress from the host erythrocyte by its capacity to bind cytoskeletal spectrin (61). Its unexpected expression in late-stage gametocytes suggests that, if expressed as a protein, it may also play a role in gametocyte egress from the host erythrocyte upon entry into the mosquito midgut, a previously undefined role for this gene that needs to be further investigated. This implies that single-cell analyses can be used to analyze highly heterogeneous cell populations and uncover unexpected expression patterns in rare cells, revealing novel functional roles of known proteins, such as MSP1.

Together, our data have presented the first single-cell analysis and comparison of male and female gametocytes of P. falciparum. These data have clearly elucidated the expression of these 87 gametocyte-specific genes in 89 gametocytes and have defined the gender identity of individual parasites. In addition, we have validated the male- and female-specific genes in individual gametocytes and have uncovered some unexpected findings, including the expression of MSP1 in late-stage gametocytes. These data provide the rationale of using single-cell approaches to dissect sexual differentiation and identify the most robust markers that can be used to isolate male or female parasites at particular stages. We expect that such single-cell analyses of malaria parasites will likely reveal important biological insights that have been masked by population analyses.

## MATERIALS AND METHODS

### Parasite strain and culture.

P. falciparum strain 3d7a, a gametocyte-producing clone of 3d7, was obtained from the Malaria Research and Reference Reagent Resource Center (MR4). Parasites were cultured at 2% hematocrit in human B+ erythrocytes at 3% O_2_/5% CO_2_ according to standard procedures using RPMI 1640 medium supplemented with 0.5% AlbuMAX II (62, 63).

### Gametocyte culture and purification.

To obtain synchronous cultures, parasites were treated with d-sorbitol twice, approximately 48 h apart during the ring stage (64). On the day after the second d-sorbitol treatment, with the parasitemia between 6 and 10%, 50 mM *N*-acetylglucosamine was added to the culture to eliminate asexual parasites (5). This was day 0. Treatment with 50 mM *N*-acetylglucosamine continued for 72 h, with daily medium changes, and gametocyte production was monitored with Giemsa-stained blood smears. On day 5 and day 9, mid- and late-stage gametocytes were separated from early gametocytes and uninfected erythrocytes by using a 40/70% Percoll density gradient, collecting the gametocytes from the 40/70% interface. To eliminate all uninfected erythrocytes, the sample collected from the Percoll gradient was run through a magnetically activated cell sorting (MACS) LS column, yielding pure gametocyte populations (5). The gametocytes were pelleted by centrifugation and resuspended in 100 µl of complete malaria media. For late-stage gametocyte collection, it may be necessary to remove contaminating trophozoites before single-cell collection. We removed rings and early gametocytes 16 h prior to single-cell capture by collecting gametocytes from a 40/70% Percoll density gradient and reseeding them in culture.

### Single-cell capture and cDNA synthesis.

To determine the viability and concentration of the gametocytes, 10 µl of gametocytes was mixed with 10 µl of trypan blue stain (0.4%) and loaded onto a Countess Automated Cell Counter (Thermo Fisher Scientific). Viable gametocytes at a concentration of 2.9 × 10^5^ to 3.7 × 10^5^ cells/ml (30 µl) were mixed in a 3:2 ratio with C1 Suspension Reagent (Fluidigm) (20 µl) before being loaded onto a C1 Single-Cell Auto Prep integrated fluidic circuit (IFC) for Preamp (5 to 10 µm). Reagent mixes were prepared according to the Fluidigm protocol (65) with the exception of the lysis final mix, which included DNase I. DNase I (Ambion) (1.4 µl) was added to 12.6 µl of Single-Cell Lysis Solution. From this, 12.75 µl was added to the lysis final mix, which also included 4.35 µl of C1 Lysis Plus Reagent (Fluidigm) and 0.9 µl of RNA Standard 1:100 dilution. For the RNA standard, spikes 1, 4, and 7 were used and were prepared according to the manufacturer’s instructions.

Primers were obtained from either Delta Gene Assays or Eurofins Genomics (see Table S1 in the supplemental material). Forward and reverse primers combined at a 100 µM concentration were pooled to a final concentration of 500 nM before being used in the preamp final mix, prepared according to the Fluidigm protocol.

The IFC was primed and loaded with 7 µl of cell mix according to the manufacturer’s instructions, using the STA: Prime 1784x and STA: Cell Load 1784x scripts. Once the cells were loaded, each well of the IFC was checked for the presence of a gametocyte under a microscope. Harvest reagent, lysis final mix, RT final mix, and Preamp final mix were then added to the IFC according to the Fluidigm protocol, and the IFC was loaded into the C1 system, running the STA: Preamp 1784x script overnight. Amplified products were collected and diluted the following morning, with 3 µl of amplicons diluted in 20 µl of C1 DNA Dilution Reagent (Fluidigm).

Controls were prepared in parallel with the C1 single cell capture. These controls included positive and negative controls (no-template and no-reverse transcriptase controls). RNA was obtained by using the RNeasy Plus Micro Kit, combining 20 µl of cell mix (2.9 × 10^5^ to 3.7 × 10^5^ cells/ml) with 330 µl of Buffer RLT Plus. Samples were homogenized by vortexing for 1 min. The lysate was then transferred to a gDNA Eliminator spin column, and RNA was obtained according to the kit protocol. After RNA was obtained, “Appendix A: Run the Tube Controls” was followed, beginning with step 8, replacing 1.0 µl of washed cells with 1.0 µl of RNA where noted. For the no-reverse transcriptase control, water was added in place of the Single Cell SuperScript reverse transcriptase (RT) (Life Technologies).

### Biomark HD qPCR.

Reagents were prepared according to Appendix B of the *Fluidigm Real-Time PCR Analysis User Guide* (66), skipping the procedures for preamplification and exonuclease treatment and starting with “Preparing Sample Pre-Mix and Samples.” Because of the fast ramp rate of the Biomark HD system, Sso Fast EvaGreen Supermix with Low ROX (Bio-Rad) was used for qPCR. The 96.96 Dynamic Array IFC was primed and loaded according to Fluidigm protocol, using the Prime (136x) and Load Mix (136x) scripts, respectively. After loading, the chip was placed into the reader and the Data Collection Software was used to set up the cycling settings. The application, reference, and probe settings were gene expression, ROX, single probe, and EvaGreen. The GE Fast 96x96 PCR+Melt v2.pcl protocol file was used, which includes 30 PCR cycles and a melting curve.

### Data analysis.

The Fluidigm Real-Time PCR Analysis Software was used to initially process the single-cell qPCR data and eliminate assays that failed. The default quality threshold cutoff of 0.65 was used to flag potential artifacts. The baseline correction field was set to linear, and the *C_T_* threshold method was set to Auto (Global). The processed qPCR results were then exported for use in downstream analyses.

The single-cell gene expression was analyzed using the SINGuLAR Analysis Toolset 3.0. The FluidigmSC library was used in R to perform outlier identification with the identifyOutliers() command. This produced a FluidigmSC expression file, which was used to perform automatic analysis with the autoAnalysis() command. The limit of detection (LoD) value was set at 24 according to the Fluidigm SINGuLAR Analysis Toolset (67, 68). The autoAnalysis() command runs principal component analysis (PCA) and unsupervised hierarchical clustering, although it does not normalize the data because there is no conclusive way to analyze burst-like expression for single cells (Fluidigm SINGuLAR Analysis Toolset [67]). Similar results for unsupervised hierarchical clustering were obtained using various filtering criteria and clustering algorithms (see Fig. S1 in the supplemental material).

To generate the dot plots, the single-cell *C*_*T*_ data were set above the detection limit on a log scale. Log_2_ expression values were obtained by subtracting the *C*_*T*_ for each gene from the LoD. If the *C*_*T*_ was greater than the LoD or the gene was not expressed in a cell, then the log_2_ expression value was zero. The averages of all male and all female single-cell log_2_ expression values were used to calculate the mean fold change in gene expression. The Mann-Whitney test was used to determine whether expression was significantly different among populations. The error bars represent the mean with the standard deviation. Dot plots are ordered by PCA gene score; the topmost left gene is the most informative when separating single cells.

For Pearson correlation clustering analysis, the –Δ*C*_*T*_ was first taken relative to the average of the RNA Standard spike-ins. These gene expression values were centered by the mean and then clustered by complete linkage analysis on both genes and arrays using Gene Cluster 3.0. A custom Perl script was generated to perform calculations of Pearson correlation coefficients based on all individual gene expression values from 90 single cells. Each coefficient represents the linear correlation between two gene expression values in a given sample population. All Pearson coefficients were clustered by the centroid linkage method in Gene Cluster 3.0 to generate the output heatmap.

### RNA fluorescent *in situ* hybridization.

Ten milliliters of a day 9 gametocyte culture was lysed with 0.15% saponin to isolate single gametocytes. Briefly, parasites were pelleted and then washed with phosphate-buffered saline (PBS) twice before being resuspended in at least 1 ml of 0.15% saponin. The parasites were then vortexed for 1 min before being placed on ice for 20 min. The cells were centrifuged at 2,100 × *g* for 12 min at 4°C and washed once in PBS. After centrifugation, these cells were fixed in 1 ml of 4% formaldehyde for 30 min. The tube was vortexed halfway between the incubation to resuspend the cells. “Appendix A: Sample Preparation Procedure for Suspension Cells” of the QuantiGene ViewRNA ISH Cell Assay (69) (Thermo Fisher Scientific) was then followed after the fixation step. Thirty microliters of poly-d-lysine (Sigma) was used to coat microscope slides, and a hydrophobic barrier was drawn. Cells were baked on the slides at 50°C for 20 min. The assay procedure then continued with the rehydration step. The probes used were Plasmodium falciparum PF3D7_0406200–Pfs16 (VF4-6000578), PF3D7_1475500–CCp1 (VF1-6000581), PF3D7_1031000–P25 (VF1-6000732), PF3D7_1311100 (VF6-6000730), PF3D7_1325200 (VF6-6000580), and PF3D7_1113900–MAPK2 (VF6-6000731). Mounted samples were cured overnight with protection from light and were viewed using a DeltaVision Elite microscope with a 60×/1.25-numerical-aperture phase-contrast oil objective. Images were taken with a CoolSNAP HQ2 high-resolution charge-coupled-device (CCD) camera with the Cy5, Alexa Fluor 594 (AF594), and fluorescein isothiocyanate (FITC) filters used to detect Cy5, Cy3, and FITC, respectively. Pfs16 was used to mark gametocyte-specific cells for cell counts in Fiji. These gametocytes were then counted for their expression of male and female markers in a merged image.
