# Supplementary material for: Single-Cell Analysis Reveals Distinct Gene Expression and Heterogeneity in Male and Female Plasmodium falciparum Gametocytes
Source: mSphere. 2018 Apr 11;3(2):e00130-18. doi: 10.1128/mSphere.00130-18 (PMC5909122; doi:10.1128/mSphere.00130-18)
Supplement: TABLE S1 [file sph002182509st1.pdf]

Supplementary Table 1: Primers used in Biomark single-cell qPCR

Gene ID	Old Gene ID	Gene Description	Forward Primer	Reverse Primer
PF3D7_1246200	PFL2215w	actin I	AGAGCTGCTCCAGAAGAACA	GTGTCATCCTCTCACGATTTCC
PF3D7_1475500	PF14_0723	CCp1, LCCL domain-containing protein	CCAAAATGTTCCCCAGAACC	AGGCGGATTTCCATTCCAA
PF3D7_1455800	PF14_0532	CCp2, LCCL domain-containing protein	AGCTATGATGGTAACGAACCA	TCATGAATAGCATGTCTCATATTAACC
PF3D7_0621100	MAL6P1.216, PFF1020C	conserved *Plasmodium* protein, unknown function	GACCATATCCAATTGGTCCATTCA	TCCAGTGGCTATTCTACCTGAC
PF3D7_0618500	MAL6P1.242, PFF0895W	MDH, malate dehydrogenase	TCGGAGCAATTGTTGGAGAA	TGGTATACCTGGGACTACATCA
PF3D7_0622800	MAL6P1.201, PFF1095W	leucine--tRNA ligase, putative	GGACCAGAATTGCCTTAGCA	AATAGCACTATTTGCCGTATCAGTA
PF3D7_0621400	MAL6P1.213, PFF1035W	Pf77	GATGTACCTGTATGTAAATATGTTCCC	TGTACAGCGGGATATGGAAC
PF3D7_0104400	MAL1P1.35, PFA0225w	4-hydroxy-3-methylbut-2-enyl diphosphate reductase	CGAAAATGCTAGAGATCCAAACA	CCGTTTCTATAGCCCGACTAA
PF3D7_0111400	MAL1P2.39, PFA0550w	GEXP19	ATTTCTTGTATCAATGGAGAGAAACA	CAATTGCAAAATGCTTGGTCATA
PF3D7_0513700	MAL5P1.137, PFE0680W, PFE0685w	PSOP12, secreted ookinete protein, putative	GTCGTGCTAGTAAGGATTCTGGTA	GCTTTTGGCGTGTGGGTAATA
PF3D7_1407000	PF14_0067	CCp3, LCCL domain-containing protein	GAAATTGCCCTCATGAAACACA	TCACCTCCAACACATCCAAC
PF3D7_0816800	MAL8P1.76	DMC1, meiotic recombination protein DMC1, putative	AGAGGATGAAGGGATAAAGGAAC	TGTCTGCAGCGTTTATACCTA
PF3D7_1325200	PF13_0144	lactate dehydrogenase, putative	AATGGCGGGTGTTCTTCATA	TCTCCATGAGCTCCTATCACA

PF3D7_1113900	PF11_0147	MAPK2, mitogen-activated protein kinase 2	GGTATAGGGCACCAGAGCTTA	GCAAATATACAACCAGTCGACCATA
PF3D7_1216500	MAL12P1.159, PFL0795c	MDV1, male development gene 1	GAGTGTTCTTCAACATCTCCAAC	AATCACCTGCTTTTGCTCCTA
PF3D7_1228300	MAL12P1.274, PFL1370w	NEK1, NIMA related kinase 1	TACCTATATGGCGATGCAGAAA	GCGCCTGTTATATTCGCTAC
PF3D7_0823500	PF08_0033	IMC1i, inner membrane complex protein 1i, putative	GCACGTTCGGTTACAGGAA	GTTGCAGGTCGTACTGATACAA
PF3D7_1025000	PF10_0244	formin 2, putative	TGCTCATGCTCAGAATGGTAAC	ACAATATCGAAGCTGGGTAAACAA
PF3D7_1030900	PF10_0302	P28, 28 kDa ookinete surface protein	CTTCATCTAACGGAGGAGGAAA	ACGTGTGTTAGGGTTACTCC
PF3D7_1031000	PF10_0303	P25, 25 kDa ookinete surface antigen precursor	CTGAAATGTGACGAAAAGACTGTA	GCGTATGAAACGGGATTTCC
PF3D7_1031600	PF10_0310	GEXP15, conserved *Plasmodium* protein, unknown function	ACAAAACGCGAATTCGAAGCA	CCATTTCAGTTGCCAATGTATGTCTA
PF3D7_1102500	PF11_0037	GEXP02, *Plasmodium* exported protein (PHISTb), unknown function	ACATGGAGGTGAATCAAAGGTA	TGCTTCTACTCATACCAGAATCC
PF3D7_1122900	PF11_0240	dynein heavy chain, putative	GTGACTTCAGACAGGGGAAA	GTGCAACACCAGCTAATATCC
PF3D7_1128300	PF11_0294	PFK11, ATP-dependent 6-phosphofructokinase	TTACTGGAGGAAATATGTTCCCAAC	GTGAGAAGTAGTTCGGTACTTAACC
PF3D7_1129900	PF11_0310	MFR5, major facilitator superfamily-related transporter, putative	CAAACTGGGCTCAGTGGATA	GTCCATTTGCACATGCTGAA
PF3D7_1311100	PF13_0060	meiosis-specific nuclear structural protein 1, putative	ACGAATATGCTCGAGAACGAA	TCATCTGACACGTTCTGTATCC

PF3D7_1339900	PF13_0218	ABCB5, ABC transporter B family member 5, putative	TACACAACATATAACACATGGTCAAC	CCCCTACACCTAGTCCTAACA
PF3D7_1407800	PF14_0075	plasmepsin IV	GTGGTTCCGAATTTGATGGTATCC	TCAACAACAACCGGATCAATAGAAC
PF3D7_1429600	PF14_0275	GEXP03, conserved *Plasmodium* protein, unknown function	CGGCATCAGAAGAGTGTGTT	TGTCTTATGCAAGACTTTGTAAGGT
PF3D7_1430800	PF14_0287	conserved *Plasmodium* protein, unknown function	TTGAAGTACCAATACCTGCACAA	GCTTGGATGCGTTTGATATTCC
PF3D7_1431100	PF14_0290	conserved *Plasmodium* protein, unknown function	AGTACCAGTCAATGTGTATGTACC	CTGGTTCCCCTTTTGCCTTA
PF3D7_1437200	PF14_0352	ribonucleoside-diphosphate reductase large subunit, putative	TGCAGACAGGGGTATTTTCA	GTAAGCTCCCGTTTTCAATCC
PF3D7_1458000	PF14_0553	FP1, cysteine proteinase falcipain 1	GAAAAGGGTATAGTACATGAGCCAAAA	CAACACTTGCAAAAGCCCAAC
PF3D7_1461800	PF14_0588	conserved *Plasmodium* protein, unknown function	GATGCTGAAGACGATGTTGAC	ACGTTTCATATCTACATGGGGATTA
PF3D7_1474900	PF14_0717	CITH, trailer hitch homolog, putative	TTGCGCCATCTAATGAAGTG	GCTGGATCGTCTGGGATATT
PF3D7_0315600	MAL3P5.20, PFC0680w	zinc finger protein, putative	CATGCCCTCTTTTGGAACTCAA	TGACATCCACGAGTCCATTCA
PF3D7_0320800	MAL3P7.12, PFC0915w	DOZI, ATP-dependent RNA helicase DDX6	AACAGCAGCTTTTGCTATACCC	TTCTCGCGTGGGTACCAAA
PF3D7_0717500	PF07_0072	CDPK4, calcium-dependent protein kinase 4	AGATGGGTCAGGTAAAATATCAGCAA	CCAACATTCTGAGCTGACATCAC
PF3D7_0517400	MAL5P1.174, PFE0870w	FACT-L, FACT complex subunit SPT16, putative	GACGAGTATGAGGTGGACGAA	TGCCAAGCTTTCGTCTTCAC
PF3D7_1302100	PF13_0011	G27/25, gamete antigen 27/25	AGGACGCAGCAGCAAGAATA	GGTGTTACGGGTAAAGCAGGTA

PF3D7_0904800	PFI0235w	RPA1, replication protein A1, small fragment	GGTCGAGTGTCTATAGCAAATCC	CCATCATCTTTCACTTCATGTATTTCAC
PF3D7_1205600	MAL12P1.56, PFL0280c	conserved *Plasmodium* protein, unknown function	GATTCCGATGTGGACGAACA	CGTTCAGCTGCTACGTCATA
PF3D7_1250100	MAL12P1.479, PFL2405c	G377, osmiophilic body protein G377	ACGACCACACGAACCAGAAA	TTCCGGTTCAGGTTCTCTGAC
PF3D7_1431500	PF14_0294	MAPK1, mitogen-activated protein kinase 1	TCGATGGTATAGAGCTCCTGAA	CTCCCATGATACAACCTAAGGAC
PF3D7_1346700	PF13_0247	P48/45, 6-cysteine protein	AGTGCACATATTTCATGTAACGTAC	CACCAGGACAATTTAAACCTACC
PF3D7_1113100	PF11_0139	PRL, protein tyrosine phosphatase	GCGGTAGCAGTTCATTGTGTA	GGCATCAATAGGGTCCATTCC
PF3D7_0209000	PF02_0086, PFB0405w	P230, 6-cysteine protein	CCAGCAGATACAGATGCTAACC	GGTTGGAAAAGGTCCAGATTCA
PF3D7_0208900	PF02_0085, PFB0400w	P230p, 6-cysteine protein	GTTGTGATTTTATAGACACACCATCA	GTGAGGATGCATGTGATCCA
PF3D7_1215000	PFL0725w, MAL12P1.145	Trx-Px2, thioredoxin peroxidase 2	TGACCTTTCCTCTTTTATAGGTCAGAA	TTCTGTTGGACATACGAAGGTA
PF3D7_1477400	PF14_0745	*Plasmodium* exported protein (PHIST), unknown function	TACCAGAACTCAAGCAAAGTTTTCA	GGAAGGTAGATTATTTCCCATAATGCA
PF3D7_1477300	PF14_0744	Pfg14-744, *Plasmodium* exported protein (PHIST), unknown function	ATTCTTACAGGCGCCAGAAC	CCTGTTTATTCCTCGCACGAA
PF3D7_1477700	PF14_0748	Pfg14-748, *Plasmodium* exported protein (PHISTa), unknown function	ATTTTAGGAGTCTTATGTGCTGAATT	CACTGCTCTAGGAACTACCC

PF3D7_0935400	PFI1710w	GDV1, gametocyte development protein 1	CGCAACATGCTCAAAAGTAACC	GGAGGCGGTTTGTGTTATTCA
PF3D7_1253000	PF12_0010, PFL2550w	GECO, gametocyte erythrocyte cytosolic protein	TGTTGTGTGTAGTTGCTTTTGT	TTGAACGATAACACACGTTTTCT
PF3D7_1476600	PF14_0736	*Plasmodium* exported protein, unknown function	ATGTCTCAACATGCGCTACA	CGCTAGTATTGGGTGAAGCA
PF3D7_1476500	PF14_0735	probable protein, unknown function	CGAGTCTTTTCTCGTCCTACC	TCTGATTGGATCCTCCTGGAA
PF3D7_1335000	PF13_0196	MSRP1, MSP7-like protein	GCAAGAAGGACAAACCGACAA	CGCCTGTACCTCCTGATTCAA
PF3D7_1346800	PF13_0248	P47, 6-cysteine protein	CCCCTGACTAATGTTAAGCCAAC	CCCAACGTAGGGTTGTTTATAGTAC
PF3D7_0406200	MAL4P1.61, PFD0310w	Pfs16, sexual stage-specific protein precursor	AAACGAAGAACGCAGATGATGATA	CTCCTTCGTCTCCTTCATCTGTA
PF3D7_0422000	MAL4P1.202, PFD1035w	steroid dehydrogenase, putative	GGATCAGTGAAAGATGCCATT	GGAACATGGCACTGAACTTG
PF3D7_0920000	PFI0980w	ELO3, long chain fatty acid elongation enzyme, putative	CATGGAGCTTTACGGTGGAT	TCGTGTGTGTATTTTTCCTGTTTT
PF3D7_1472900	PF14_0697	dihydroorotase, putative	CACTTAAGGCAAGGCGACAT	CGTGTTCGGCATCACTAAAA
PF3D7_0927100	PFI1330c	conserved *Plasmodium* protein, unknown function	TGCTTGTAGACTTGTTGGCTCT	TTTATGTTCCCGTTCGCTTT
PF3D7_0215000	PF02_0141, PFB0685c	ACS9, acyl-CoA synthetase	ACGTTTATAACAGGCGGAGGTA	TCCAGTCGTTTCAGTTAATCCA
PF3D7_1236200	MAL12P1.348, PFL1750c	conserved *Plasmodium* protein, unknown function	AAATTTCCCGTTGATGTCCA	AGTTCCGTGATGTTGTTCCA
PF3D7_0525000	MAL5P1.249, PFE1245w	zinc finger protein, putative	AATACAAACGCCACATCGAA	CTTGCTTCATTCCCTTGGTT

PF3D7_1305200	PF13_0026	ApiAP2, AP2 domain transcription factor, putative	TGGTTTAATACAAAACAGCAGGAA	CTAGCTGCTTCAAAATTTCGTTTT
PF3D7_0604100	MAL6P1.44, PFF0200c	SIP2, SPE2-interacting protein	GGAATTTGAAATGCAGCAAAA	TTCTCCATACCATCCCCTTT
PF3D7_1143100	PF11_0442	AP2-O, AP2 domain transcription factor AP2-O, putative	CCAACCTGATGAACAACCTG	TTCCACCTTACCGCTATTCC
PF3D7_1408200	PF14_0079	AP2-G2, AP2 domain transcription factor AP2-G2, putative	TCGTGTTAGTTGGAAGGATCAA	TCCTGCTTTAGTGGTCCATTTT
PF3D7_1317200	PF13_0097	ApiAP2, AP2 domain transcription factor, putative	TTGATAAATCTGGGAGGAGGTG	TATTTAGCTTGTGCCTGACCAA
PF3D7_0404100	MAL4P1.40, PFD0200c	AP2-SP2, AP2 domain transcription factor AP2-SP2, putative	GCCAGCACGTTTGAACCTTA	AGTTGTCCCCTTTTCAATGC
PF3D7_0420300	MAL4P1.192, PFD0985w	ApiAP2, AP2 domain transcription factor, putative	GATGGGATTCCACGAAAAGA	ATTACTAGCGGGTGCTGACG
PF3D7_0801900	PF08_0133, MAL8P1.154	conserved *Plasmodium* protein, unknown function	TACCACCAACAGCGATACCA	GAAGAAACGGATGGAGACCA
PF3D7_0206300	PF3D7_0206400, PF02_0059, PF02_0060, PFB0279W, PFB0280W	AROM, pentafunctional AROM polypeptide, putative, pseudogene	AGGACAGGAAACAGCATACG	CACTAAGAACTTTCCATCTTTTCCA
PF3D7_1466200	PF14_0631	conserved *Plasmodium* protein, unknown function	TGTGGTGAATGCGGTTAATG	TGCGTTATTTCCTTCATTTGC
PF3D7_0422300	MAL4P1.205, PFD1050W	alpha tubulin 2	TGGTGGTGATGATGCCTTTA	GTCAACGACGGTGGGTTCTA
PF3D7_0717700	PF07_0073	serine--tRNA ligase, putative	TGGAACAATGGTAGCTGCAC	ATGTATGGGCGCAATTTTTC

PF3D7_0202000	PF02_0020, PFB0100C	KAHRP, knob-associated histidine-rich protein	TTTTAGTATGGGTTTTGAAGTGCTC	TGTTGCTTTTGTGCTAAAGTTCTC
PF3D7_0930300	PFI1475W	MSP1, merozoite surface protein 1	AACTGGCCTCGAAGCTGATA	GGAACCATTTGCTGAATGTG
PF3D7_1107800	PF11_0091	ApiAP2, AP2 domain transcription factor, putative	GGAAATGTGGGTTCTACACCA	CCCCAGAAGAGACTGCAGAA
PF3D7_1363800	MAL13P1.320	GEX1, nuclear fusion protein, putative	GATTAGCGCAAACATCCTTGT	TTCGCTGAAATCGTTCTTGA
PF3D7_1014200	PF10_0139	HAP2, male gamete fusion factor HAP2, putative	ATGTCGTACTGTCGTTTATGGAGA	CCTCAGACCTTAAAACCTTTAACC
PF3D7_1426500	PF14_0244	ABCG2, ABC transporter G family member 2	AAGTGGTGGTCAAAGAAAAAGATT	AATCCACTTGTAGGTTCATCCATT
PF3D7_1222600	MAL12P1.217, PFL1085W	AP2-G, AP2 domain transcription factor AP2-G	GATGGTGGGAAAATGGAAGA	TAGCCATTCTCAATGCACCA
PF3D7_1114000	PF11_0148	dynein light chain Tctex-type, putative	TGTTGATGACGTGGAGCAAT	CGTTCCAAAGAGACTTGGATG
PF3D7_1413200	PF14_0131	conserved *Plasmodium* protein, unknown function	TGGATACGAGCAGCAGTTGT	TGGTTGTTATGGAATGCTTGG
PF3D7_0719200	MAL7P1.100	NEK4, NIMA related kinase 4	ACCCCTTTTCATTCAACCAA	TGCTCTGTAACTTGGTTCTCG
PF3D7_0525900	MAL5P1.258, PFE1290W	NEK2, NIMA related kinase 2	AAGCTTTCGTTGAGGATTGTG	TTCTCAGGGATGGGAGTTTC
PF3D7_0518700	MAL5P1.187, PFE0935c	PUF1	CAAACGAGGAGTACAAAACAAAAA	GCAAAATCCTACAACCCGTTT
PF3D7_0417100	MAL4P1.160, PFD0825c	PUF2	TCATCAAAAAGATTGTCAACGA	TGCATCATAAAATTACCATAGCAG
