# Supplementary material for: Single-Cell Analysis Reveals Distinct Gene Expression and Heterogeneity in Male and Female Plasmodium falciparum Gametocytes
Source: mSphere. 2018 Apr 11;3(2):e00130-18. doi: 10.1128/mSphere.00130-18 (PMC5909122; doi:10.1128/mSphere.00130-18)

A. Present in 50%
Mean-centered, at least one observation>1
Average Linkage

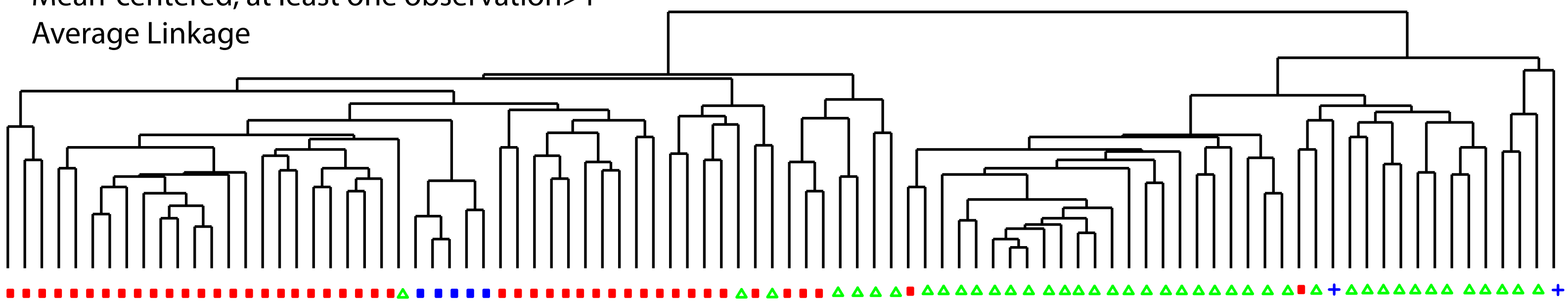

B. Present in 50%
Mean-centered, at least one observation>1
Centroid Linkage

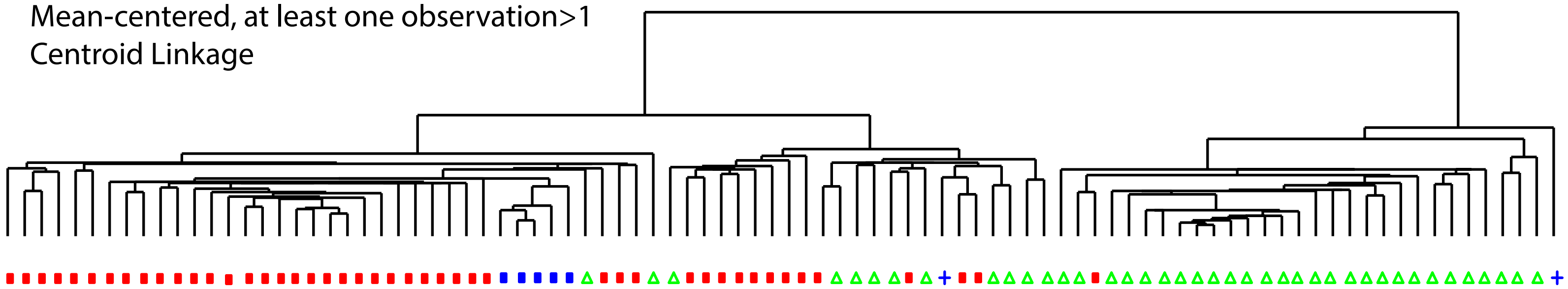

C. Present in 70%
Mean-centered, at least one observation>1
Complete Linkage

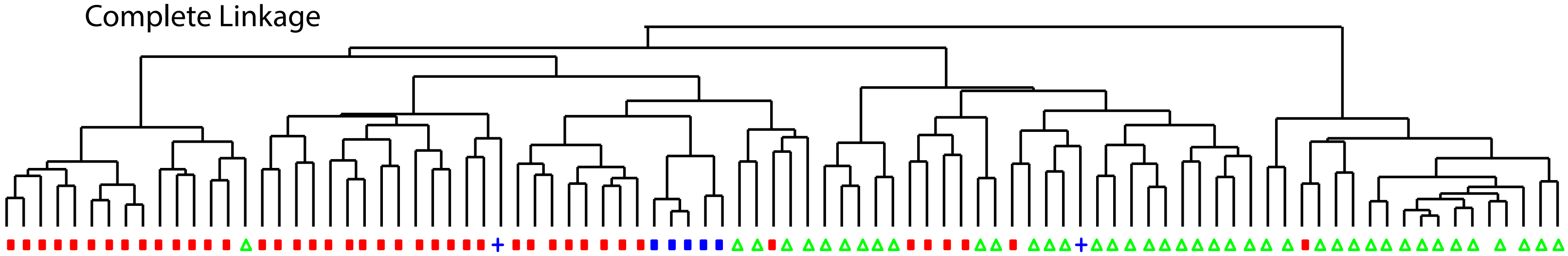

D. Present in 60%
Mean-centered, at least one observation>1
Average Linkage

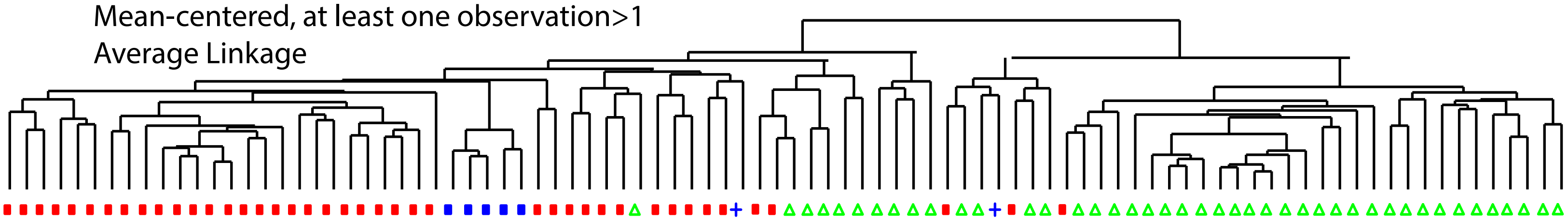

Supplement: FIG S1 [file sph002182509sf1.pdf]
