# Supplementary figures and images for: Single-Cell Analysis Reveals Distinct Gene Expression and Heterogeneity in Male and Female Plasmodium falciparum Gametocytes
Source: mSphere. 2018 Apr 11;3(2):e00130-18. doi: 10.1128/mSphere.00130-18 (PMC5909122; doi:10.1128/mSphere.00130-18)

**A**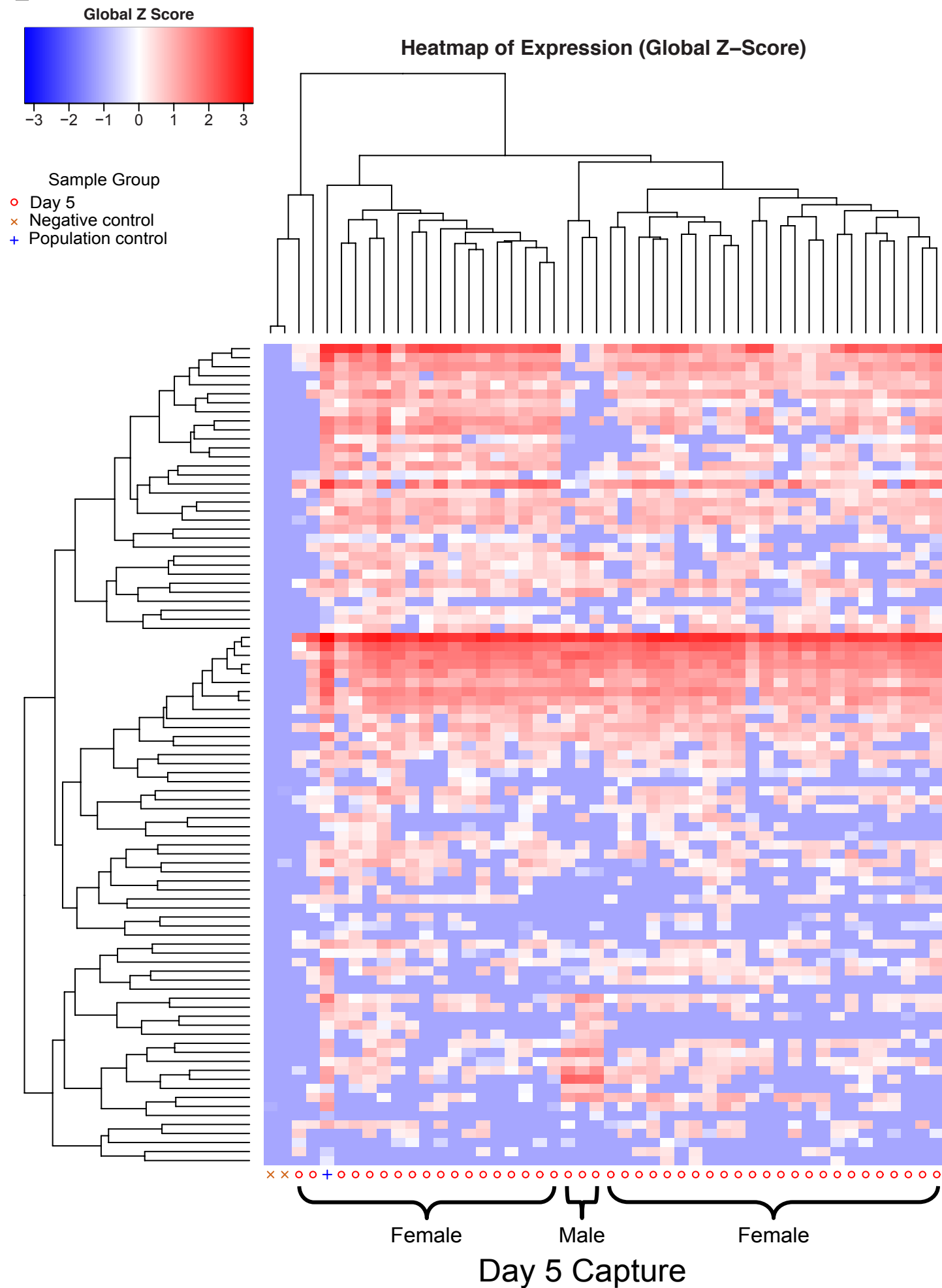**B**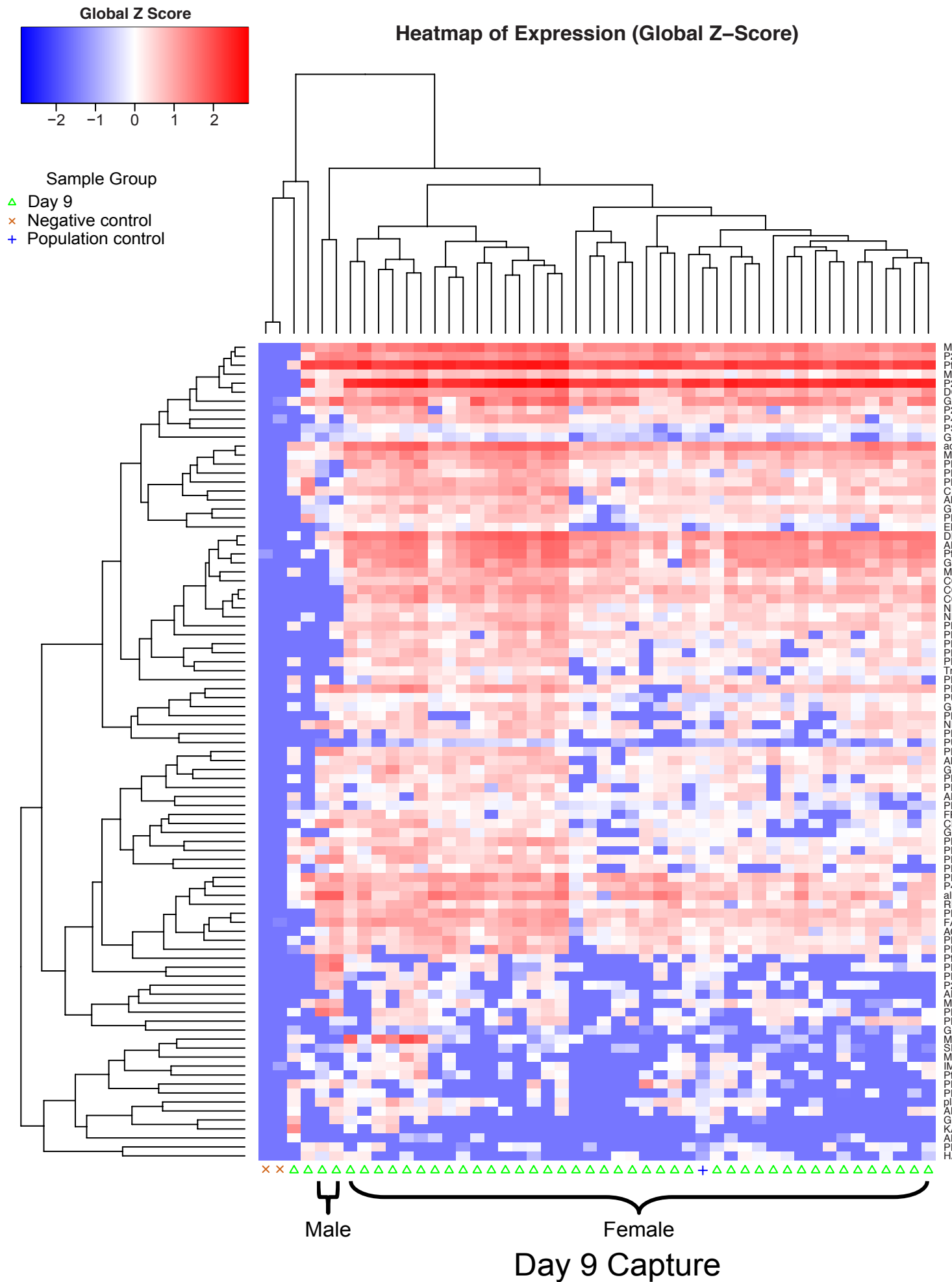

Supplement: FIG S2 [file sph002182509sf2.pdf]

A

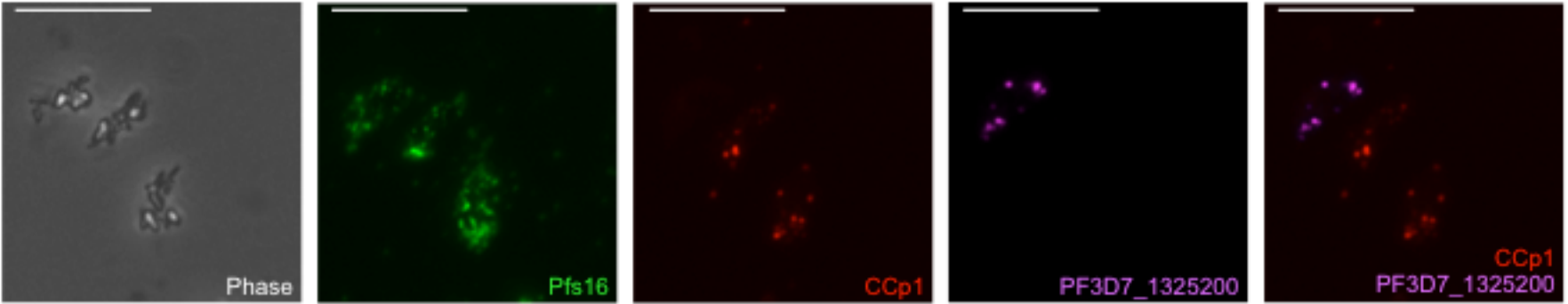

B

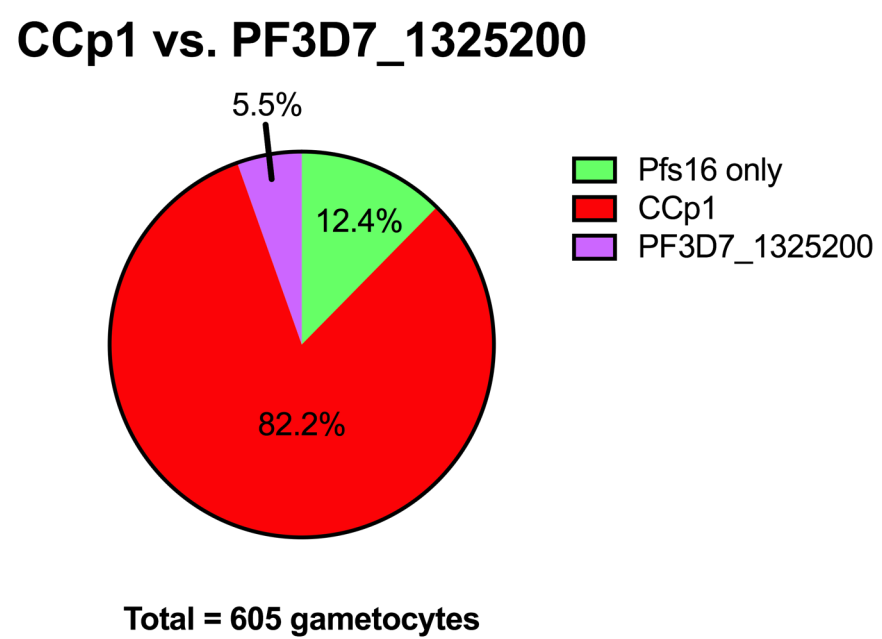

C

|      |   | PF3D7_1325200 |     |
|------|---|---------------|-----|
|      |   | +             | -   |
| CCp1 | + | 0             | 497 |
|      | - | 33            | 75  |

D

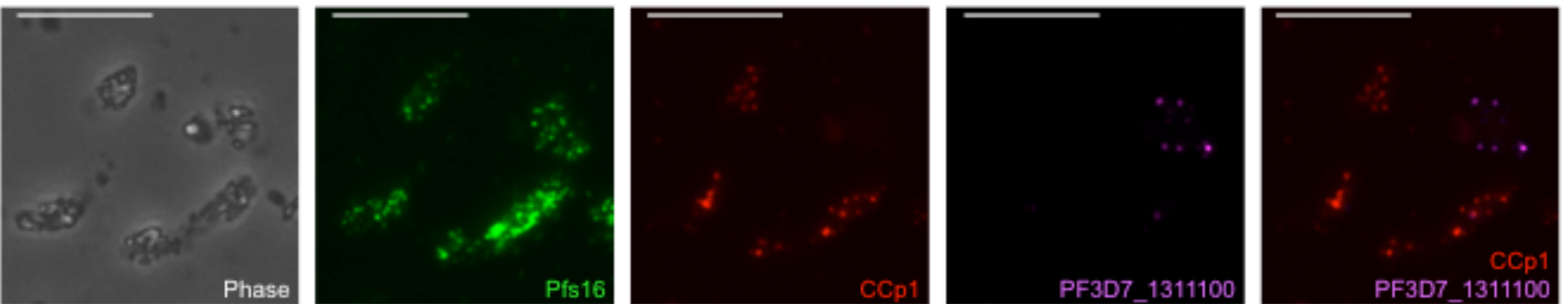

E

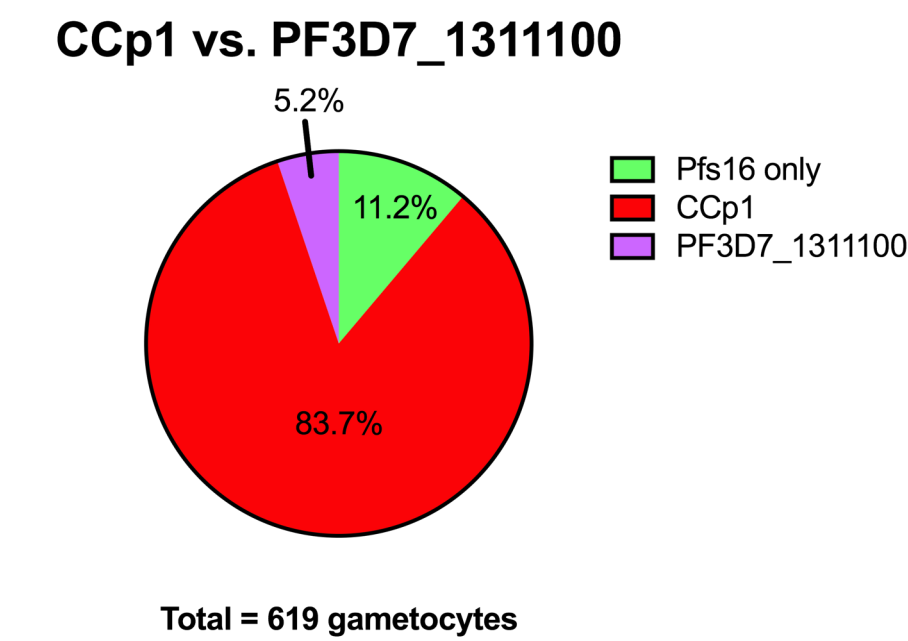

F

|      |   | PF3D7_1311100 |     |
|------|---|---------------|-----|
|      |   | +             | -   |
| CCp1 | + | 0             | 518 |
|      | - | 32            | 69  |

G

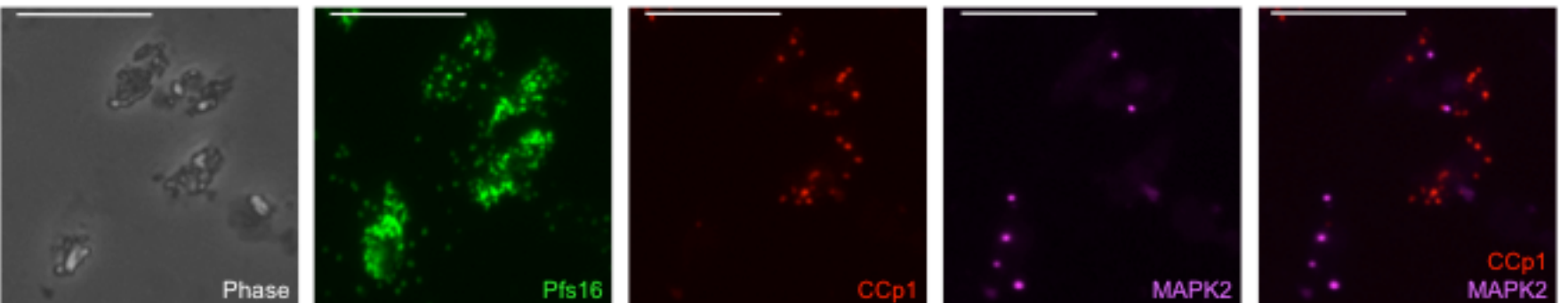

H

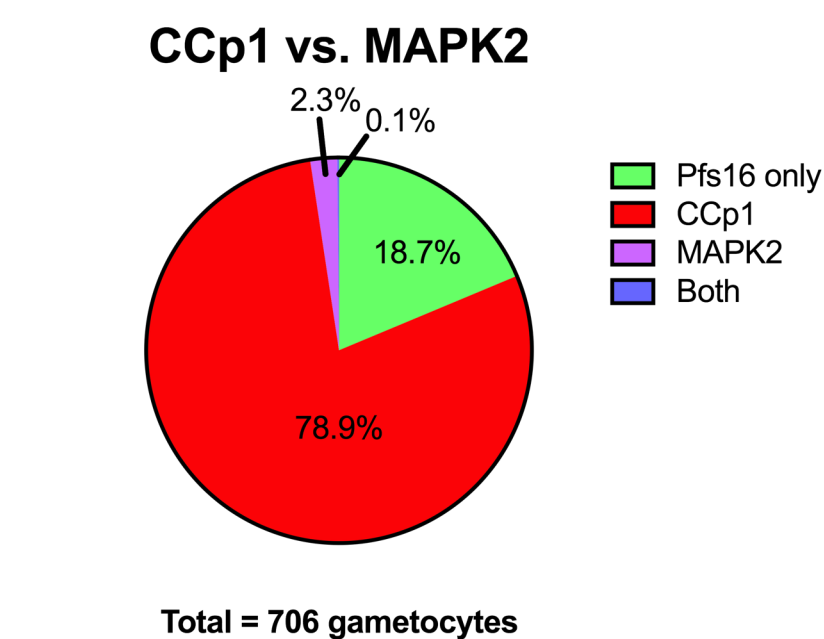

I

|      |   | MAPK2 |     |
|------|---|-------|-----|
|      |   | +     | -   |
| CCp1 | + | 1     | 557 |
|      | - | 16    | 132 |

Supplement: FIG S4 [file sph002182509sf4.pdf]
