# Supplementary material for: Single-Cell Analysis Reveals Distinct Gene Expression and Heterogeneity in Male and Female Plasmodium falciparum Gametocytes
Source: mSphere. 2018 Apr 11;3(2):e00130-18. doi: 10.1128/mSphere.00130-18 (PMC5909122; doi:10.1128/mSphere.00130-18)

Transcript levels of genes from purified male and female gametocytes

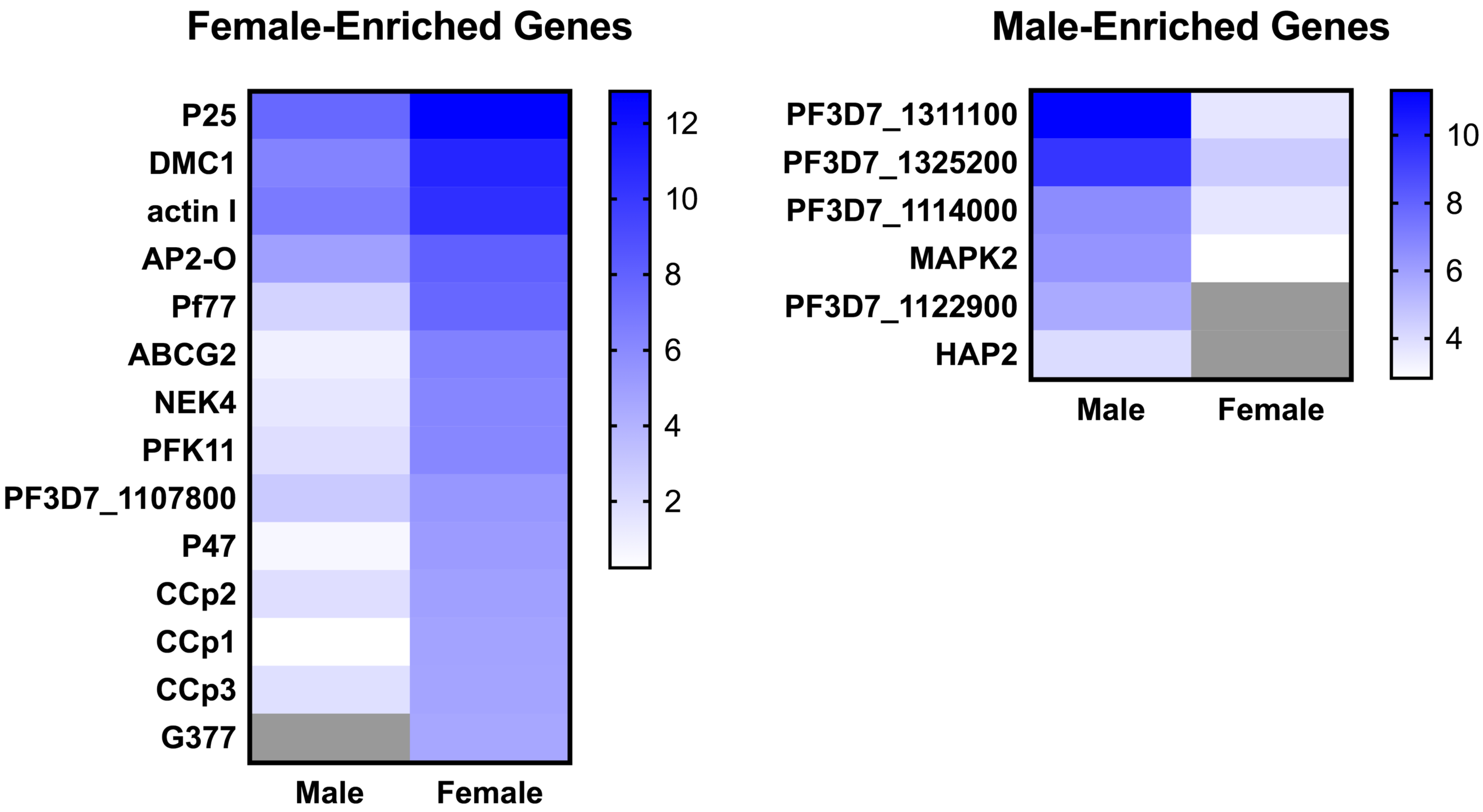

Supplement: FIG S3 [file sph002182509sf3.pdf]
